# Combining the induced pluripotent stem cell (iPSC) technology with chimeric antigen receptor (CAR)-based immunotherapy: recent advances, challenges, and future prospects

**DOI:** 10.3389/fcell.2024.1491282

**Published:** 2024-11-18

**Authors:** Mehdi Alidadi, Haniyeh Barzgar, Mohammad Zaman, Olga A. Paevskaya, Yekta Metanat, Elnaz Khodabandehloo, Vahid Moradi

**Affiliations:** ^1^ Department of Anatomy, School of Medicine, Tehran University of Medical Sciences, Tehran, Iran; ^2^ Department of Anatomical Sciences, School of Medicine, Zahedan University of Medical Sciences, Zahedan, Iran; ^3^ Department of Genetics, Islamic Azad University Tehran Medical Branch, Tehran, Iran; ^4^ Department of Infectious Diseases, Institute of Public Health named after F.F. Erisman, I.M. Sechenov First Moscow State Medical University of the Ministry of Health of the Russian Federation (Sechenov University), Moscow, Russia; ^5^ Faculty of Medicine, Zahedan University of Medical Sciences, Zahedan, Sistan and Baluchestan, Iran; ^6^ Department of Immunology, School of Medicine, Hamadan University of Medical Sciences, Hamadan, Iran; ^7^ Hematology and blood transfusion science department, School of Allied Medical Sciences, Tehran University of Medical Sciences, Tehran, Iran

**Keywords:** induced pluripotent stem cell (iPSC), chimeric antigen receptor (CAR), cGMP, cancer, immunotherapy

## Abstract

After experiencing many ups and downs, chimeric antigen receptor (CAR)-T cell therapy has reached a milestone as an anti-cancer method, as evidenced by the increasing number of clinical trials and approved products. Nonetheless, there is a real need to optimize CAR-T cell therapy and overcome its existing limitations. The importance of cellular starting material for generating CAR-T cells is undeniable, as the current personalized manufacturing approach is the main roadblock to providing a fast, affordable, and standard treatment for patients. Thus, developing an off-the-shelf CAR-T product is a leading focus in adoptive cell therapy. Several biotech companies worldwide are focused on developing an off-the-shelf CAR-T product from allogeneic sources. Induced pluripotent stem cells (iPSCs) have unique characteristics, making them highly attractive among various allogeneic sources. IPSCs can be modified with CAR, undergo other intended gene manipulations, and then be differentiated into functional hematopoietic lineages with anti-cancer activity. Moreover, iPSCs provide an unlimited cell source, simplifying the setting of a standard treatment protocol by generating a homogenous population of resulting cells and reducing batch-to-batch inconsistency. In this review, we delve into the manufacturing of iPSC-derived CAR-T (iCAR-T) cells and discuss the path and challenges of their clinical translation. We also introduce some iPSC-derived cellular alternatives to conventional iCAR-αβ-T cells, including iCAR-T cells with a limited TCR diversity, iCAR-NK, iCAR-macrophages, and iCAR-neutrophils and discuss their relative advantages and disadvantages as well as their differentiation from iPSCs in compliance with cGMP. Finally, we reviewed iPSC-derived CAR-engineered cells being evaluated in clinical trials.

## 1 Introduction

The introduction of chimeric antigen receptor (CAR)-T cells in the 1990s (Gross and Eshhar, 1992; Eshhar et al., 1993; Stancovski et al., 1993) has ushered a new era in the field of anti-cancer treatments, as evidenced by at least 11 commercially available CAR-T products and hundreds to thousands of registered clinical trials. Since the approval of Kymriah (a CD19 CAR-T product) in 2017 by the US Food and Drug Administration (FDA) for patients with B-cell acute lymphoblastic leukemia (B-ALL) (Maude et al., 2018) Five other CAR-T products have been approved by this authority, including Yescarta (a CD19 CAR-T product approved in 2017) (Neelapu et al., 2017), Tecartus (a CD19 CAR-T product approved in 2020) (FDA, 2021a), Breyanzi (a CD19 CAR-T product approved in 2021) (FDA, 2021c), Abecma (a BCMA CAR-T product approved in 2021) (FDA, 2021b), and Carvykti (a BCMA CAR-T product approved in 2022) (FDA, 2022). Other regulatory authorities have also authorized some other CAR-T products. China National Medical Products Administration (NMPA) approved two CD19 CAR-T products, including Carteyva (JW Therapeutics, 2021) and CNCT 19 (Liu et al., 2024), as well as two BCMA CAR-T products, including Fucaso (Keam, 2023) and “Zevor-Cel, CT053” (Chen et al., 2022; Fu et al., 2023). The Spanish Agency of Medicines and Medical Devices (AEMPS) has also approved ARI-001, a CD19 CAR-T cell, for adult patients with B-ALL (Ortíz-Maldonado et al., 2021).

The approved CAR-T products listed above are manufactured using T cells derived from the patient’s own peripheral blood (PB) based on a custom process. Although autologous CAR-T cell therapy has been widely recognized as an effective treatment option for patients with CD19^+^ hematologic malignancies and multiple myeloma, the extensive application of this treatment is impeded by several limitations. Most of the drawbacks of this individualized manner are attributed to its starting material, patients-derived T cells. Due to the immune-suppressive nature of cancer and anti-cancer agents, the patient’s T cells may lack the desired cellular fitness and may not be viable for *ex vivo* expansion to achieve a clinically significant quantity. The lack of appropriate cellular fitness can lead to production failure in 7% of patients with B-ALL and 25% of patients with non-Hodgkin lymphoma (NHL) (Baguet et al., 2024). Manufacturing autologous products normally takes around 2–4 weeks, another access-limiting factor in cases of urgent needs (Shah et al., 2023). Moreover, treatment failure can occur in up to 40% of patients with B-ALL and up to 50% of patients with B-cell lymphoma (Baguet et al., 2024). In these cases, re-administration of CAR-T cells may be required. However, this is hindered by the limited number of cells obtained. Even in the case of successful manufacturing and treatment, the high cost of commercially available autologous products (more than $375,000 for FDA-approved products) imposes a high-cost burden on patients (Moradi et al., 2023). The difference between cellular fitness and composition in different patients results in the batch-to-batch inconsistency of final products, making it difficult to set a standard drug dosing and treatment protocol (Sommermeyer et al., 2016; Cuffel et al., 2022). Finally, while a high safety profile is considered a significant advantage of autologous CAR-T cell therapy over allogeneic therapy, recent reports of disease relapse after CAR-T cell therapy due to product contamination by leukemic cells have raised concerns in this regard (Ruella et al., 2018).

Based on the mentioned limitations of autologous CAR-T cell therapy, there is a real-time demand for an appropriate cellular starting material to manufacture an off-the-shelf, homogenous, affordable, scalable, and reproducible product. In the following sections, we delve into the induced pluripotent stem cells (iPSCs) as a reliable cell source for CAR-based immunotherapy.

## 2 IPSCs and their merits over other cell sources

The issues mentioned above have moved the focus of several biotech companies worldwide to provide an appropriate master stock as starting material for CAR-T cell manufacturing. In recent years, allogeneic CAR-T cell therapy has emerged as an alternative to address the current issues of autologous treatment. Nearly 100 clinical trials using allogeneic CAR-T cells have been designed in recent years, and the released data of some of these trials are promising (Moradi et al., 2023). Third-party donors, umbilical cord blood, and pluripotent or multipotent stem cells are the primary cellular sources for these trials. Each source has relative advantages and disadvantages that must be considered in its use (Table 1).

**TABLE 1:** Comparison of CAR-T cells derived from different sources.

	Autologous CAR-T	Third-party donor-derived CAR-T	Cord blood-derived CAR-T	HSC-derived CAR-T	IPSC-derived CAR-T
Cellular fitness of the final product	Unfavorable	Favorable	Favorable	Favorable	favorable
Batch-to-batch consistency	Inconsistent	Inconsistent	Inconsistent	Inconsistent	Consistent
Manufacturing capacity	Limited	Limited (from a single donor)	Limited (from one unit)	Limited (from a single donor)	Potentially unlimited
Immunogenicity	Low	High	Relatively Low	High	High
Multiplexed gene editing	Possible but challenging	Possible but challenging	Possible but challenging	Possible but challenging	More straight forward
Safety concerns	CRS, ICANS	GvHD, CRS, ICANS	GvHD, CRS, ICANS	GvHD, CRS, ICANS	GvHD, CRS, ICANS, Teratoma
Accessing time	2–4 weeks	Readily available	Readily available	Readily available	Readily available
Re-administration	Difficult	Possible	Possible	Possible	Possible

Generating healthy donor-derived genome-edited CAR-T cells is the most widely utilized approach in clinical trials to develop an off-the-shelf CAR-engineered cell product. In this method, genome editing tools such as CRISPR/Cas9 are employed to reduce the immunogenicity of allogeneic cells and prevent the risk of inducing graft versus host disease (GvHD) in allogeneic HLA-mismatched CAR-T cell therapy (Lonez and Breman, 2024). Although the use of healthy donor-derived CAR-T cells could address most of the bottlenecks of autologous therapy, the batch-to-batch inconsistency remains unsolved. Different donors have different cellular compositions and immune characteristics, which can lead to variations in batches derived from different donors. On the other hand, due to the limited volume of blood donated by each donor and the limited expansion capacity of T cells, each donor-derived product is sufficient for only a limited number of patients (Moradi et al., 2023). Another reliable T cell source is umbilical cord blood (UCB), which is rich in T cells with naive and less differentiated phenotypes. Due to the high expansion capacity and longevity, transfusion of UCB-derived CAR-T cells provides a sustainable anti-tumor activity. Low immunogenicity, low risk of GvHD occurrence, and the presence of private/public UCB banks worldwide are other advantages of UCB-derived CAR-T cells (Liu D-D. et al., 2022). Despite the promising results of UCB-derived CAR-modified cells in several preclinical and clinical studies (Yu et al., 2023; Liu et al., 2020), it should be noted that each cord blood unit yields about 10^8^ CAR-T cells (Yu et al., 2023) that can be enough for only a single adult patient.

Utilizing pluripotent/multipotent stem cells as master stock cell sources could be another alternative. While ethical issues restrict the therapeutic application of embryonic stem cells (ESCs), the use of hematopoietic stem cells (HSCs) and induced pluripotent stem cells (iPSCs) for the generation of hematopoietic lineages have gained attention in recent years. HSCs have been efficiently engineered with CAR and differentiated toward CAR-T and CAR-NK cells with therapeutic efficacy. However, expanding primary HSCs while retaining their stemness is the main obstacle in their application for generating CAR-T cells on an industrial scale (Arias et al., 2021; Tang and Zhang, 2024).

The advent of iPSCs by Dr. Yamanaka in 2006 (Takahashi and Yamanaka, 2006) was a revolution in the field of cell-based treatments since their stemness and differentiation capacity are on par with ESCs while avoiding ethical issues of ESCs. Theoretically, iPSC can be generated by introducing reprogramming factors (Oct3/4, Sox2, Klf4, and c-Myc) to any somatic cells and can differentiate into any desired cell lineage. iPSCs can be generated using patient-derived cells for personalized therapeutic approaches or allogeneic cells to provide off-the-shelf products. While iPSCs are very similar to ESCs in terms of surface markers, stemness, proliferation rate, morphological features, and feeder cell-dependent cultivation, they differ in transcriptional and epigenetic profiles (Scalise et al., 2021). In recent years, iPSCs have emerged as a reliable source for generating CAR-T cells. In the following sections, we discuss the process of generating iPSC-derived CAR-T cells (iCAR-T cells).

## 3 Generation of iCAR-T cells

CAR-T cell therapy in both autologous or allogeneic settings mainly relies on the use of αβ-T cells, the most well-known subset of T cells, and the dominant subpopulation of T cells in peripheral blood. Here the key requirements regarding the generation of clinical-grade CAR-αβ-T cells from iPSCs are briefly highlighted.

### 3.1 Generation of iPSCs

Reprogramming the somatic cells to iPSCs is the first step toward manufacturing iCAR-T cells. To generate iPSCs for clinical applications, three key factors that can affect reprogramming efficiency should be initially considered: i) the initial cell type, ii) The method of introducing the reprogramming factors into the cells, and iii) cell culture protocol.

#### 3.1.1 Initial cell type

Since the first report of iPSC generation using fibroblasts, different cell types from different tissues have been used to generate iPSCs, so today, almost all cell types can be reprogrammed into iPSCs. Nonetheless, the most frequently used cells to generate iPSCs include skin fibroblast, keratinocytes, and peripheral blood- or cord blood-derived mononuclear cells. Generally, irrespective of initial somatic cell type, reprogramming is a cumbersome process in which, in ideal conditions, its efficiency is just up to 4% (Poetsch et al., 2022). It has been revealed that the reprogramming efficiency is highly affected by the age and type of the initial cells. Although iPSCs can be generated using senescent cells, reprogramming juvenile cells is more efficient and faster than senescent cells (Lapasset et al., 2011). It has also been shown that the reprogramming efficiency of cord blood- and peripheral blood-derived CD34^+^ cells is considerably higher than that of terminally differentiated peripheral blood CD34^negative^ mononuclear cells (PBMCs) (Jha et al., 2021). The results of various studies have shown that the reprogramming efficiency of keratinocytes is 100 times higher than that of fibroblasts. iPSCs generated from keratinocytes are produced in a faster time and are more similar to ESCs (Klingenstein et al., 2020).

The above-mentioned findings indicate the influence of initial cell type in the reprogramming efficiency. When the final purpose of iPSC generation is its differentiation toward T-cell lineage for adoptive cell therapy, the choice of initial cell type can affect the final result. In various studies, iPSC-derived T (iT) cells have been successfully generated using reprogramming of either T or non-T cells (Themeli et al., 2013; Netsrithong et al., 2020; Montel-Hagen et al., 2019; Nishimura et al., 2013; Maeda et al., 2020). However, it has been found that somatic memory of the initial cell type can affect the differentiation of iPSCs into the intended lineage and can also affect some of the characteristics of the resulting cells. Somatic memory means that iPSCs retain the epigenetic and transcriptomic signatures of their origin cell and tissue, which can affect their behavior (Khoo et al., 2020). For example, it has been revealed that compared to iPSCs generated from fibroblasts and keratinocytes, iPSCs reprogrammed from T cell clones (T-iPSCs) retain the VDJ rearrangement pattern of their parent T cells and that the resulting T cells express the same T-cell receptor (TCR) as the parent T cells (Netsrithong and Wattanapanitch, 2021). It can be considered a safeguard in the autologous settings since T-iPSC-derived T cells will not express an autoreactive TCR because of the removal of autoreactive T cells in the thymus. In contrast, in the cases of using non-T cells as starting material, the random VDJ recombination of TCR can give rise to the generation of autoreactive T cells. In allogeneic settings, whether T cells are used as starting materials or non-T cells, the resulting T cells would be alloreactive, which is associated with the risk of inducing graft versus host disease (GvHD). In this regard, knocking out of the intrinsic TCR of iCAR-T cells or the use of T-cell clones with defined TCR repertoire as starting cellular material for the generation of iPSCs could be a solution to circumvent the risk of GvHD (Mazza and Maher, 2021).

Although it is demonstrated that the initial somatic cell type can affect the iPSC differentiation and behavior of the resulting cells, the significance and amount of these influences have not yet been completely clarified. There is a real-time need for comparative studies to determine the effect of initial cell type in the differentiation of iPSCs toward T cells and the efficacy of generated CAR-T cells.

#### 3.1.2 Introducing the reprogramming factors

Historically, the first attempt to introduce reprogramming factors into the somatic cell was made using a retroviral vector (Takahashi and Yamanaka, 2006). Nonetheless, the random integration profile of integrating viral (lentiviral-retroviral) vectors carries the risk of insertional mutagenesis, which can lead to tumorigenesis by altering the expression pattern of tumor suppressor genes or oncogenes (Moretti et al., 2022). In recent years, integrating transposon vectors (such as sleeping beauty and piggyBac) have emerged as safe alternatives to retroviral/lentiviral vectors because of their safe random integration profile (Metanat et al., 2024). Nonetheless, recent cases of leukemic transformation in a clinical trial of piggyBac-mediated CAR-T cells have raised worries about their safety (Schambach et al., 2021). On the other hand, transgene integration-based methods are not suitable for clinical applications since the integrated transgenes may not become transcriptionally silenced over time, and their permanent expression leads to tumorigenesis (Shao and Wu, 2010).

Given that only transient expression of reprogramming factors is required for cell reprogramming, in recent years, several non-integrating protocols have been used in this regard. While all of these non-integrating approaches circumvent the risks related to the permanent expression of reprogramming factors and avoid the risk of insertional mutagenesis, they are different regarding safety, efficiency, and compliance with current good manufacturing practice (cGMP). Adenoviral vectors were the first non-integrating vectors used to generate iPSCs (Sridharan et al., 2009). The cargo capacity of this vector is ∼8kb, which allows delivery of all four factors in the form of a single polycistronic transgene. Even with this, adenoviral vector-mediated generation of iPSCs suffers from low reprogramming efficiency (about 0.0006%), cumbersome manufacturing process, and requiring multiple steps of cell transduction (Shao and Wu, 2010; Karami et al., 2022). Temperature-sensitive Sendai viral vectors have been efficiently used to introduce reprogramming factors into the somatic cells and production of iPSCs without integrating foreign genetic materials within the host genome. Temperature-sensitive mutations in this vector enable removing viral vector components and reprogramming factors at nonpermissive temperatures (Ban et al., 2011). Nonetheless, due to the lack of a cGMP-compliant regent, this approach is not applicable to clinical approaches (Abraham et al., 2018).

Episomal expression of reprogramming factors is another option to generate transgene-free iPSCs. Plasmids are the most known episomal vectors that can be manufactured under cGMP with significantly lower cost than clinical-grade viral vectors (Metanat et al., 2024). However, their transfection rate is cell-type dependent and may be toxic for cells due to their large size. Moreover, their bacterial backbone may cause transgene silencing. Bacterial backbone-free minicircles are suitable alternatives to plasmids since their small size and lack of bacterial elements increase the efficiency of transfection and transgene expression (Jia et al., 2010; Narsinh et al., 2011). Origin of replication (oriP)/Epstein-Barr nuclear antigen-1 (EBNA1) containing plasmids can replicate within the cells and sustain transgene expression for a while that is enough for reprogramming, so circumvent the need for multiple transfections (Kumar et al., 2018). Nonetheless, they have shown low reprogramming efficiency in various studies. Another drawback of these vectors is that they may exert long-term episomal expression of reprogramming factors (Yu et al., 2009). Excision competent/integration defective (exc + int-) variants of sleeping beauty \ piggyBac vectors can also be used for transgene-free iPSCs (Moretti et al., 2022).

Although the mentioned episomal vectors are non-integrating, the risk of their insertion into the host genome is possible (Mulia et al., 2021). DNA-free reprogramming approaches allow the transient activity of reprogramming factors and remove the risk of insertional mutagenesis. In this regard, mRNA-, miRNA-, protein, and small molecules-based approaches have been developed to generate iPSCs. among the non-integrating approaches, the mRNA-based method has the highest reprogramming efficiency. By bypassing the need for DNA transcription, mRNA-mediated reprogramming takes less time than DNA-based approaches. While low *in vivo* stability is the main bottleneck of the mRNA-based method, some modifications, such as phosphatase treatment and replacing cytidine and uridine with 5-methylcytidine and pseudouridine, respectively, significantly improve the stability of mRNA molecules. As the mRNAs have a low life span, repeated mRNA transfection to complete the reprogramming is crucial (Warren and Lin, 2019). In this regard, the use of self-replicating RNAs can yield a more durable expression of reprogramming factors and remove the necessity for multiple transfections (Yoshioka et al., 2013). In recent years, several microRNAs (miRNAs) (such as mir-200c, mir-302, and mir-369 families) have also been discovered to be involved in the pluripotency of stem cells. Transfection of these miRNAs has been successfully used to reprogram both mouse and human cells into iPSCs (Yoshioka et al., 2013; Omole and Fakoya, 2018).

One of the safest reprogramming methods for clinical purposes is the use of proteins, which mediates the reprogramming process without the risk of inducing genetic scars. Proteins can be fused to other compounds, such as polyarginine, to increase their transfection into the cells. Nonetheless, this method suffers from low efficiency and requires a long time to reprogram. Production and purification of reprogramming factors are other difficulties of these methods (Omole and Fakoya, 2018). There are also several small molecules with the ability to reprogram somatic cells to iPSCs. Certain mixtures of these small molecules have been successfully used to reprogram somatic cells. small molecules can also be combined with other methods to synergize their reprogramming efficiency. Generally, these small molecules mediate their reprogramming roles via three main mechanisms: affecting cellular signaling pathways, alteration of epigenetic profile, and metabolic changes. Compared to other approaches this method offers a safer and cost-beneficial approach that simplifies large-scale manufacturing of iPSCs (Karami et al., 2022).

#### 3.1.3 IPSC culture considerations

While traditionally iPSCs are manufactured in xenogeneic feeder cell-based culture conditions, the use of xeno-free culture protocols is vital for their clinical translation. Although human-derived feeder cells can also be used for iPSC culture, their preparation is time-consuming and labor-intensive. On the other hand, It has been revealed that they can disturb the maintenance of iPSCs (Nakagawa et al., 2014). In recent years, xeno-free and feeder-free approaches have been developed to generate iPSCs under cGMP. Since iPSCs are anchorage-dependent, feeding the extracellular matrix (ECM) to sustain their survival and pluripotency is required in feeder-free protocols (Horiguchi and Kino-oka, 2021). These ECMs can be classified as undefined, semi-defined, and fully defined. Undefined matrices are crude ECM extracts (animal-derived or xeno-free) that are a mixture of ECM proteins with undefined ratios. Although they support the proliferation and pluripotency of iPSCs in culture, their lot-to-lot variation may affect the reproducibility of IPSCs (Hagbard et al., 2018). Semi-defined matrices are proteins (such as laminin, collagen, fibronectin. And vitronectin) purified from natural sources. Difficulties in the purification of these proteins in large amounts, the risk of affecting the phenotype and behavior of iPSCs due to the presence of other biological contaminants, and batch-to-batch variability are the main bottlenecks of these products (Hagbard et al., 2018; Wondimu et al., 2006). In recent years, the development of xeno-free, contaminant-free recombinant matrix proteins such as recombinant laminin, fibronectin, and vitronectin with fully defined composition put an additional level on the safety of clinical-grade iPSCs and iPSC-derived products. They are commercially available in research-grade and clinical-grade forms with low batch-to-batch inconsistency (Kasai et al., 2020; Valamehr et al., 2011).

In recent years, scientists have also developed feeder-free cell culture mediums, which are a milestone for cGMP‐compliant iPSC manufacturing. Essential 8™ and MTeSR™ are the most frequently used mediums that have been successfully used for scale-up manufacturing of iPSCs. They are capable of supporting the expansion of iPSCs for more than 50 passages without affecting their karyotype, pluripotency, and differentiation capacity (Ghaedi and Niklason, 2019; Hayal and Doğan, 2022). The culture media should be changed daily to support iPSCs’ growth and prevent their differentiation. It has been shown that enzymatic dissociation and mechanical dissociation methods can lead to genetic instability of iPSCs (Garitaonandia et al., 2015; Maitra et al., 2005; Lefort et al., 2008; Spits et al., 2008; Steichen et al., 2019) Therefore, the use of non-enzymatic passaging methods such as EDTA-based dissociation is recommended (Beers et al., 2012). In the case of ESC culture, It has been revealed that Rho‐associated protein kinase (ROCK) inhibitors can prevent dissociation‐induced apoptosis without affecting the pluripotency and differentiation capacity of ESCs (Rivera et al., 2020). Nonetheless, the effect of ROCK inhibitors on iPSCs is controversial. While some results indicate the effectiveness of adding ROCK inhibitors to iPSC culture media (Claassen et al., 2009; Matsumoto et al., 2022), others indicate that the metabolism and differentiation capabilities of iPSCs are altered by exposure to ROCK inhibitors (Jiang et al., 2022; Vernardis et al., 2017; Maldonado et al., 2016).

### 3.2 Characterization and quality control of the generated iPSCs

Several in-process quality controls must be performed to ensure the safety, pluripotency, and differentiation capacity of generated iPSCs. The efficiency of the reprogramming may vary and result in different partially- or fully-reprogrammed iPSCs. The emerged colonies can be initially selected based on their ESC-like morphology, positive alkaline phosphatase staining, and doubling time kinetics (Karami et al., 2022). iPSCs can be characterized using the analysis of the expression of pluripotency markers, including SSEA3, SSEA4, OCT4, Tra-1-60, Tra-1-81, Oct3/4, Sox2, and Nanog. Expression of these markers can be analyzed at the protein level using flow cytometry and immunohistochemistry or at the mRNA level using qPCR and RNA sequencing (Baghbaderani et al., 2015). Combinational evaluation of at least one surface marker and one intracellular marker is recommended (Sullivan et al., 2018). The loss of the construct used for reprogramming must also be evaluated (Jha et al., 2021). The reprogramming process, serial subculturing, and freeze/thaw make iPSCs prone to acquire chromosomal abnormalities and mutations. Genomic stability of iPSCs must be confirmed using methods such as G-band karyotyping assay, multicolor FISH (M-FISH) analysis, chromosomal microarray analysis, Whole genome sequencing, whole exome sequencing, and cDNA expression analysis (Steichen et al., 2014; Baghbaderani et al., 2016). multi-lineage differentiation capacity of generated iPSCs can be evaluated using *in vitro* differentiation assays (embryoid body formation and directed differentiation assay) and *in vivo* teratoma formation assay (Sadeqi Nezhad et al., 2021). Microbiological sterility assays must be performed to detect contaminations by bacteria, *mycoplasma*, viruses, and endotoxins, according to the FDA guidance document 21 CFR 610.12 (Sullivan et al., 2018; FDA, 2024). Sterility assays should be performed at various stages and should not be limited to the final product (Sullivan et al., 2018).

### 3.3 CAR modifications and other gene manipulations

#### 3.3.1 CAR engineering

While the integrating viral (lentiviral and gamma retroviral) vectors are mainly used for introducing CAR to the target cells, in recent years, several integrating (transposon vectors) and non-integrating (mRNA, plasmids, minicircles, nanoplasmids, and nanocarriers) non-viral vectors and delivery methods have been developed to remove the safety concerns, exorbitant cost and cumbersome production process of viral vectors (Metanat et al., 2024). To generate iCAR-T cells, CAR transgene can be introduced to iPSCs or to already differentiated iT cells. CAR engineering at both levels has relative advantages and disadvantages. At the level of iT cells, both integrating and non-integrating vectors can be used to engineer iT cells with CAR. The use of non-integrating vectors and delivery methods removes the concern of insertional oncogenesis. Additionally, when the target antigen has a shared expression between normal and malignant tissues (more prominent in solid tumors), the transient expression of CAR can prevent on-target off-tumor toxicities. Nonetheless, Given the short duration of CAR expression, multiple transfusions of CAR-engineered cells may be required to complete the eradication of the tumor (Metanat et al., 2024; Nethi et al., 2023).

For direct engineering of iPSCs, it makes more sense to use integrative vectors because CAR expression is lost during differentiation and expansion if non-integrative vectors are used. Lentiviral, retroviral, and transposon vectors (sleeping beauty, piggyBac, Tol2, and TcBuster) can yield stable expression of CAR (Metanat et al., 2024). Nonetheless, the random integration profile of these vectors raises concerns about the risk of insertional oncogenesis. Recently, reports of changes in the cellular behavior of lentiviral-mediated CAR-T cells (Shah et al., 2019; Fraietta et al., 2018) and leukemic transformation of piggyBac-mediated CAR-T cells (Micklethwaite et al., 2021) raise these worries. Moreover, randomly integrated CAR may become silent during the differentiation of iPSCs toward hematopoietic lineages. In an elegant work conducted by Kong and colleagues, it was demonstrated that iPSCs could be effectively engineered using either lentiviral or piggyBac vectors. However, the expression of CAR has been downregulated during the differentiation of CAR-iPSCs into iCAR-macrophages (Kong et al., 2022). In this regard, the site-directional insertion of CAR using gene editing tools into the safe genomic harbors within the host cell genome can prevent insertional oncogenesis and CAR silencing over time (Kimura et al., 2019). Programmable nucleases such as zinc finger nucleases (ZFNs), transcription activator-like effector nucleases (TALENs), and CRISPR/Cas9 are targeted nucleases capable of introducing a double-strand break (DSB) at desired genomic sites. Non-homology end joining (NHEJ) and homology-directed repair (HDR) are two cellular intrinsic repair pathways that are responsible for the repair of introduced DSB. NHEJ is an error-prone mechanism that ultimately results in disruption of target genes due to the random insertion/deletion mutations. In contrast, HDR precisely repairs the cleaved site using a homologous template. If a template consisting of the desired sequence, which lies within the homology arms, is introduced to the DSB site, HDR uses this template to repair the cleaved site, ultimately resulting in the insertion of the desired sequence within the damaged site (Moradi et al., 2024). Several safe genomic harbors for targeted CAR insertion have been discovered in recent years, enabling stable and uniform expression of CAR. These genomic harbors are included AAV1, TRAC, TRBC, PCD1, TET2, CCR5, and ROSA26 loci. The size of the CAR transgene allows its delivery to the DSB site using an adeno-associated viral (AAV) vector that is safe for clinical applications (Dabiri et al., 2023).

#### 3.3.2 Gene editing to prevent graft versus host disease (GvHD)

While iPSC technology can be used in either autologous or allogeneic settings, the focus of iPSC-derived CAR-T cell therapy is mainly on its allogeneic applications to overcome the limitations of the autologous CAR-T cell therapy. Nonetheless, the allogeneic nature of CAR-T cells poses two challenges to this method. Due to the diversity of intrinsic TCR of iCAR-T cells and HLA mismatch between recipients and donors, the infused iCAR-T cells may cause GvHD and immunologically be rejected by the patient’s immune responses. Although the use of fully or partially HLA-matched donor cells can reduce the immunogenicity of iCAR-T cells and the risk of GvHD, this approach is hindered by the logistical challenges of finding an HLA-matched donor (Cichocki et al., 2023). In the past decade, gene editing tools, specifically the CRISPR/Cas9 system, have paved the way for utilizing allogeneic CAR-T cells safely and efficiently. In the CRISPR system, the target site recognition is mediated by the single guide (sg) RNA, making its re-purposing easier than ZFNs and TALENs, whose re-purposing to new target sites requires laborious protein engineering processes. Moreover, performing a multiplex genome-editing strategy (editing two or more genes simultaneously in the same cell) is more straightforward using CRISPR/Cas9 (Moradi et al., 2024). Gene editing tools have been successfully used to remove endogenous TRC from the surface of CAR-T cells. This can be achieved by disrupting either the *TCR alpha constant (TRAC)* or *TCR betta constant (TRBC)* genes, which encode the TCR alpha and beta chains, respectively (Madison et al., 2022). Additionally, the disruption of the *TRAC/TRBC* genes can be coupled with the site-directional insertion of CAR at the disrupted site. Using this method, CAR is expressed under endogenous TCR transcriptional elements that result in uniform expression of CAR between the cells and remove the risk of insertional oncogenesis (Eyquem et al., 2017).

#### 3.3.3 Gene editing to prevent graft rejection

**FIGURE 1:**
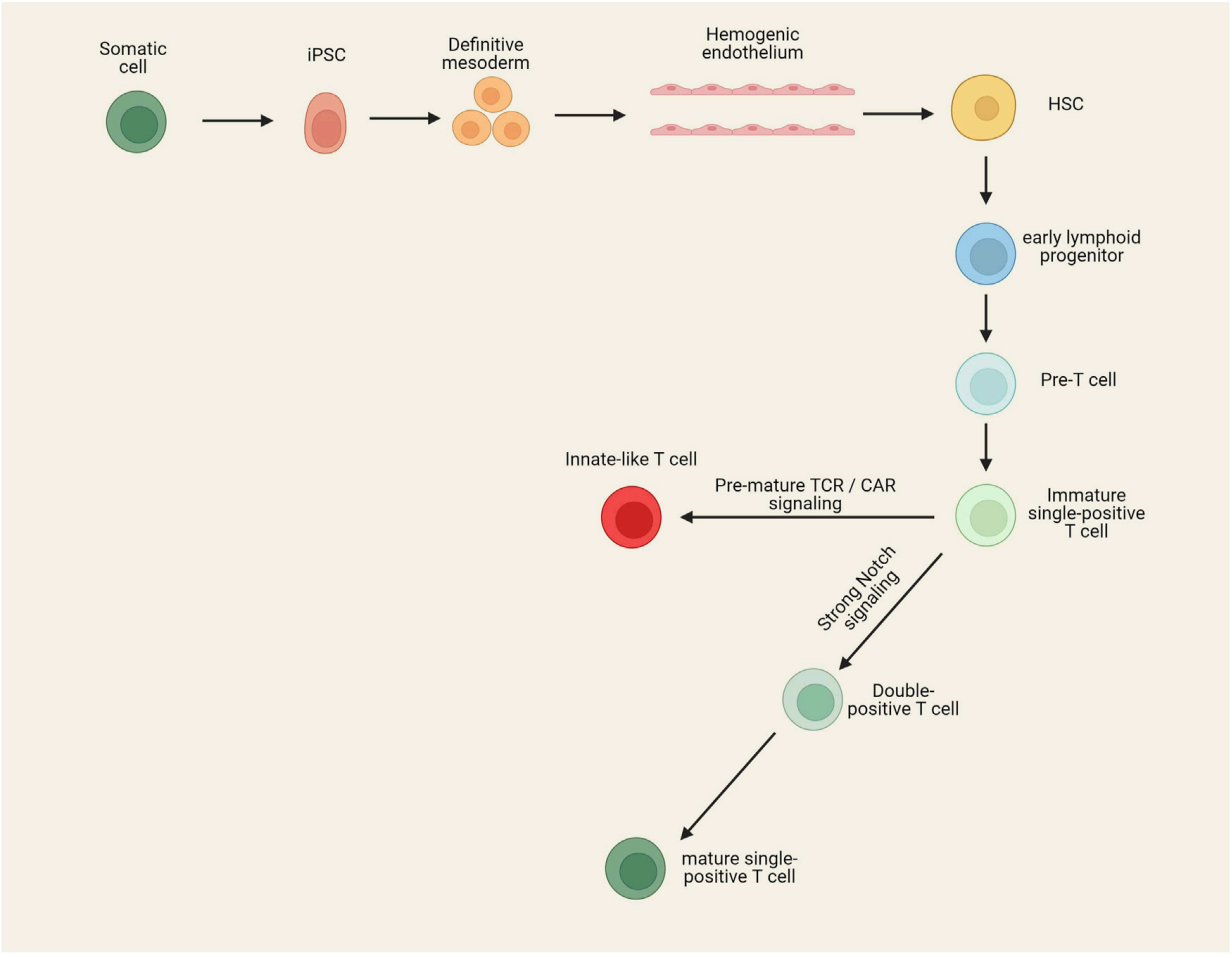
Gene editing strategies to develop hypoimmunogenic iCAR-engineered cells with reduced risk of GvHD. Removing the expression of TCR through the genetic ablation of TRAC/TRABC genes can prevent the occurrence of GvHD. Reducing the immunogenicity of allogeneic cells can be performed using different strategies. Host CD8^+^ and CD4^+^ T cell-mediated rejection can be blunted by disruption of MHC-I (knocking out of the B2-M gene) and MHC-II (knocking out of CIITA and RFX genes). Insertion of an NK inhibitory ligand and disruption of CD155 can prevent NK cell-mediated rejection of alloimmune cells. Insertion of an alloimmune defense receptor (ADR) can also prevent graft rejection. An alternative method to prevent graft rejection is based on the disruption of CD52 or deoxycytidine kinase (dck), which allows the depletion of host lymphocytes by the administration of exogenous agents. Insertion of an off-switch receptor or suicide gene makes it possible to deplete the infused cells in case of severe adverse effects.

Programmable nucleases can also be utilized to reduce the immunogenicity of infused iCAR-T cells (Figure 1). Surface removal of MHC-I and MHC-II molecules can prevent rejection of iCAR-T cells by host CD8^+^ and CD4^+^ T cells, respectively. The MHC genes are highly polymorphic, so their direct targeting is extremely difficult. MHC-I molecules can be eliminated by disrupting β2 microglobulin (β2M), a shared non-polymorphic chain between MHC-I molecules. MHC-II molecules can also be eliminated from the cell surface by targeting CIITA and RFX genes, two regulators of MHC-II expression (Wang B. et al., 2021; Mishra and Qasim, 2024).

While the disruption of MHC-I and MHC-II molecules blunts both CD4^+^ T and CD8^+^ T cell-mediated rejection of iCAR-T cells, the absence of MHC-I molecules can cause host natural killer (NK) cell-mediated rejection of iCAR-T cells (Kagoya et al., 2020). Several strategies have been developed to blunt NK cell-mediated rejection of allogeneic cells. One of these strategies is the targeted disruption of HLA-A and HLA-B molecules but not non-canonical HLA-C molecule (Kitano et al., 2022; Xu et al., 2019). This strategy is laborious as it needs to design several sgRNAs and select iPSC colons harboring all intended edits. Nonetheless, homozygous iPSCs can make this process easier since one sgRNA can induce biallelic disruption of each of the HLA-A/HLA-B molecules (Kitano et al., 2022). Another strategy is introducing a ligand to iPSCs to promote NK inhibitory signals or suppress NK activating signals. β2m-HLA-E/β2m-HLA-G fusion proteins, siglec 7/9, and E-cadherin are among the NK cell inhibitory ligands that can be inserted into the iPSC genome to prevent NK cell-mediated rejection of iCAR-T cells (Depil et al., 2020). It has been revealed that allogeneic CAR-T cells with disruption of both MHC-I and MHC-II molecules and insertion of an NK inhibitory ligand have a prolonged *in vivo* persistence than MHC-I and -II disrupted CAR-T cells (Li W. et al., 2022).

CD155, or poliovirus receptor (PVR), is another ligand on CAR-T cells that can trigger NK cell-mediated rejection of CAR-T cells via interaction with DNAM-1, an NK cell-activating receptor. In a study by Wang et al., a combinational gene editing/engineering strategy have used for increasing iPSC-derived T cells. They combine the disruption of β2M, CIITA, and CD155 genes with the insertion of HLA-E transgene, which significantly increases the immune escape of iPSC-derived T cells (Wang B. et al., 2021). They used this combinational strategy on the basis that the HLA-E inhibitory ligand is not capable of inhibiting all host NK cells as about half of NK cells are NKG2A^negative^ (receptor of the HLA-E) (Fauriat et al., 2008). In this regard, coupling the insertion of the HLA-E ligand with the disruption of CD155 can completely blunt NK cell-mediated immune rejection of iCAR-T cells. It has also been revealed that the insertion of CD47 and PD-L1, two immune checkpoint molecules, can blunt NK cell-mediated rejection and macrophage-mediated phagocytosis of CAR-T cells (Beckett et al., 2023; Rong et al., 2014). Recently, scientists have developed a 4-1BB-specific alloimmune defense receptor (ADR) whose expression on the membrane of CAR-T cells can blunt all three CD4^+^ T cell-, CD8^+^ T cell-, and NK cell-mediated rejection of allogeneic CAR-T cells. 4-1BB is expressed on the surface of activated lymphocytes but not resting lymphocytes; thus, using this receptor, the transfused allogeneic cells can reverse the anti-graft responses of the host lymphocytes and persist *in vivo* for a longer time (Mo et al., 2021).

While a multiplex genome editing strategy makes it possible to generate hypoimmunogenic iCAR-T cells, it should be noted that this strategy is relatively cumbersome and also increases the risk of chromosomal abnormalities (Samuelson et al., 2021). In this regard, a different strategy can be used to prevent the rejection of infused cells without requiring the insertion/deletion of multiple genes. This strategy is based on the disruption of either CD52 or deoxycytidine kinase (dck), which allows the depletion of host lymphocytes by anti-CD52 monoclonal antibodies and fludarabine, respectively, without depleting iCAR-T cells (Tees et al., 2021; Valton et al., 2015).

#### 3.3.4 Off-switch receptors

CAR-T cell therapy is not without risk and is associated with several adverse effects, such as on-target off-tumor toxicities, cytokine release syndrome (CRS), and Immune effector cell-associated neurotoxicity syndrome (ICANS). Secondary tumors due to leukemic transformation of infused CAR-T cells or contamination of final iCAR-T products by iPSCs are other rare risks of iCAR-T cell therapy (Schambach et al., 2021; Wang, 2023). In this regard, adding an off-switch receptor or suicide gene into the iCAR-T cells makes it possible to deplete them by administering an exogenous agent when required. In recent years, different types of off-switch receptors have been designed. For example, co-expression of CAR and a cell surface epitope of CD20 or EGFR allows the depletion of CAR-T cells by administration of rituximab and cetuximab, respectively (Serafini et al., 2004; Wang et al., 2011). Inducible caspase 9 (iCasp9) is another off-switch receptor composed of Caspase 9 fused to a modified FK506-binding protein (FKBP1A). By systemic administration of rimiducid (AP 1903), iCasp9 becomes dimer and triggers apoptosis (Rafiq et al., 2020). It has been revealed that by administration of rimiducid, iCasp9 can eliminate more than 90% of cells in less than 30 min (Di et al., 2011). Herpes simplex virus thymidine kinase (HSV-TK) Mut 2 gene can also be used as a suicide gene in CAR-T cells, which enables the elimination of infused cells by administration of ganciclovir (Tomasik et al., 2022). Nonetheless, the viral nature of HSV-TK increases the risk of immunogenicity. Additionally, the HSV-TK-mediated depletion of infused cells is very slow and has low efficiency (Netsrithong et al., 2024).

### 3.4 Differentiation of CAR-iPSCs into iCAR-T cells

#### 3.4.1 Differentiation of CAR-iPSCs into HSCs

Differentiation of CAR-iPSCs into functional iCAR-T cells demands the meticulous coordination of specific biological processes resembling embryonic development. For this purpose, iPSCs must first differentiate into HSCs and then commit to T cells. This process involves the initial formation of definitive mesoderm, the precursor of various adult cell types, including hematopoietic cells, skeletal muscles, and cardiac muscles. Further differentiation of definitive mesoderm gives rise to a transient stage called hemogenic endothelium, where a specific type of endothelial cells can generate HSCs during endothelial to hematopoietic transition (Mazza and Maher, 2021). This complex process requires precise orchestration of various signaling pathways, transcription factors, and alteration of gene expression profile. Activin A, BMP4, Wnt3a, and CHIR99021 growth factors must be added to the iPSC culture medium to induce definitive mesoderm. These growth factors bind to their receptors on the surface of iPSCs and induce downstream signaling pathways, including Wnt/β-catenin, Activin/Nodal, and BMP pathways (Ruiz et al., 2019). These signal transductions ultimately lead to the activation of transcription factors such as Brachyury (T) and Eomesodermin (Eomes), which changes the gene expression profile necessary for the induction of KDR^+^APLNR^+^PDGFRα^low/−^ mesoderm (Tosic et al., 2019). Subsequent timed administration of growth factors encompassing VEGF and FGF elicit the activation of transcription factors such as GATA2 and RUNX1, which facilitate the differentiation to VE-cadherin (CD144)^+^ CD73^negative^ CD235a/CD43^negative^ hemogenic endothelium (Garcia-Alegria et al., 2018; Bresciani et al., 2021). By Further modulation of signaling pathways through adding vascular endothelial growth factor (VEGF), stem cell factor (SCF), thrombopoietin (TPO), FMS-related tyrosine kinase 3 ligand (FLT-3L), interleukin-3 (IL-3), IL-6, and StemRegenin 1 (SR1) hemogenic endothelium undergoes endothelial-to-hematopoietic transition, eventually resulting in the formation of HSCs (Choi et al., 2012; Zheng et al., 2023).

Differentiation of iPSCs into HSCs can be mediated using either 2D or 3D culture systems. Depending on the research goals, both of these systems can be utilized. 2D systems are more suitable for initial research because of their relatively simple and cost-beneficial process (Rao et al., 2022). The most well-known 2D culture system is based on the co-culture of iPSCs with murine stromal cell lines such as OP9, MS5, C3H10T1/2, and S17 (Zheng et al., 2023). These cell lines produce and secrete growth factors required for iPSC differentiation toward HSCs. While this method gives rise to HSCs resembling definitive hematopoiesis, xenogeneic cell lines impede their clinical use. Another 2D differentiation method is based on the seeding of iPSCs on a surface coated with extracellular matrix such as vitronectin, collagen, Matrigel, and Tenascin C. This method requires growth factors and small molecules to be added in a timed manner to direct iPSC differentiation toward HSCs. While avoiding the use of murine stromal cell lines makes this approach compliant with cGMP, large-scale production of HSCs using this method is challenging.

The use of *in vivo* or *in vitro* 3D differentiation systems resembling *in vivo* microenvironments can improve the efficiency of iPSC differentiation toward HSCs. In 2013, for the first time, it was revealed that the injection of human iPSCs into immunodeficient mice gave rise to teratoma, a kind of benign tumor with differentiated cells of all three germ layers (Amabile et al., 2013; Suzuki et al., 2013). This method provides a niche-like environment that supports the differentiation of iPSCs toward functional HSCs (Tsukada et al., 2017). Nonetheless, it is challenging to translate this technique into the clinical setting and to produce HSCs on a large scale using it. Another well-known technique to induce differentiation of iPSCs in a 3D microenvironment is based on the use of embryoid bodies (EBs). EBs are three-dimensional structures with multicellular aggregates comprising the mesodermal, ectodermal, and endodermal lineages formed by spontaneous differentiation of human pluripotent stem cells. Due to the resembling embryonic condition, differentiation of HSCs from iPSCs on EBs is more efficient than on 2D cultures (Mora-Roldan et al., 2021). While the EB-induced differentiation of iPSCs normally leads to primitive hematopoiesis, induction of definitive hematopoiesis is also possible by the timely addition of activin inhibitors and manipulation of the Wnt-β-catenin signaling pathway (Nafria et al., 2020; Garcia-Alegria et al., 2021). Reproducibility is the main bottleneck of this method since, depending on the donor of the initial somatic cell, the size and quality of EBs, media composition, and other lab-to-lab variables, the final output of this process can vary (Rao et al., 2022; Garcia-Alegria et al., 2021). As an alternative to EBs, ESC–derived sacs (ES-sacs) can also be used for *in vitro* differentiation of iPSCs in a 3D environment; nonetheless, this method also suffers from reproducibility challenges (Martin et al., 2019; Haro-Mora et al., 2020). In recent years, several organoid-like 3D differentiation systems have been developed, which indicate the superiority of 3D differentiation systems over 2D methods (Ackermann et al., 2021; Shan et al., 2021; Ma et al., 2021; Wu et al., 2023).

#### 3.4.2 T-cell commitment

In recent years, various methods have been developed to simulate the thymus environment for T-cell differentiation. The key point of all these methods is to provide Notch signaling, a critical regulator of T-cell development in the thymus. The most well-known method for T cell differentiation is based on the co-culture with stromal cells. In this method, mouse stromal cell lines such as OP9 cells are manipulated to overexpression of Delta-like 1 (Dll1) or Dll4, the human homologs of the Notch ligand, to induce the required Notch signaling in HSCs. Andrawes et al., have revealed that dll4 binds to the Notch receptor with a significantly higher affinity than Dll1 (Andrawes et al., 2013). In addition to Dll1/4, providing Flt3L and IL-7 is also necessary for the T-cell specification (Nishimura et al., 2019). As the mouse stromal cells cannot provide TCR stimulation for human T-cell progenitors, the addition of an anti-CD3 monoclonal antibody is necessary for T-cell development (will be discussed in detail in the following section) (Maeda et al., 2016). The use of artificial thymic organoids is another feeder-based approach that provides a 3D structure resembling the thymus environment for T cell development. These organoids are formed by aggregating DLL1/4 expressing MS5 mouse stromal line with human HSPCs through centrifugation (Seet et al., 2017). This method has been successfully used to generate iPSC- and ESC-derived T cells (Montel-Hagen et al., 2019). Wang et al., have revealed that the anti-tumor activity of anti-CD19 CAR-T cells generated by artificial thymic organoids is comparable to that of peripheral blood-derived CAR-T cells (Wang et al., 2022).

The use of mouse stromal cell line in the abovementioned approaches limits their clinical translation. On the other hand, efforts to use human feeder cells to develop a Xeno-free differentiation approach have not been very satisfactory (Mohtashami et al., 2013; Lapenna et al., 2013). Several feeder-free differentiation systems have been developed to overcome this limitation. In these methods, DLL1/4 becomes immobilized by fusing to the Fc domain of IgG, VCAM-1, retronectin, or microbeads (Varnum-Finney et al., 2003; Shukla et al., 2017; Iriguchi et al., 2021; Trotman-Grant et al., 2021).

### 3.5 Technical and biological hurdles for the generation of iCAR-T cells

**FIGURE 2:**
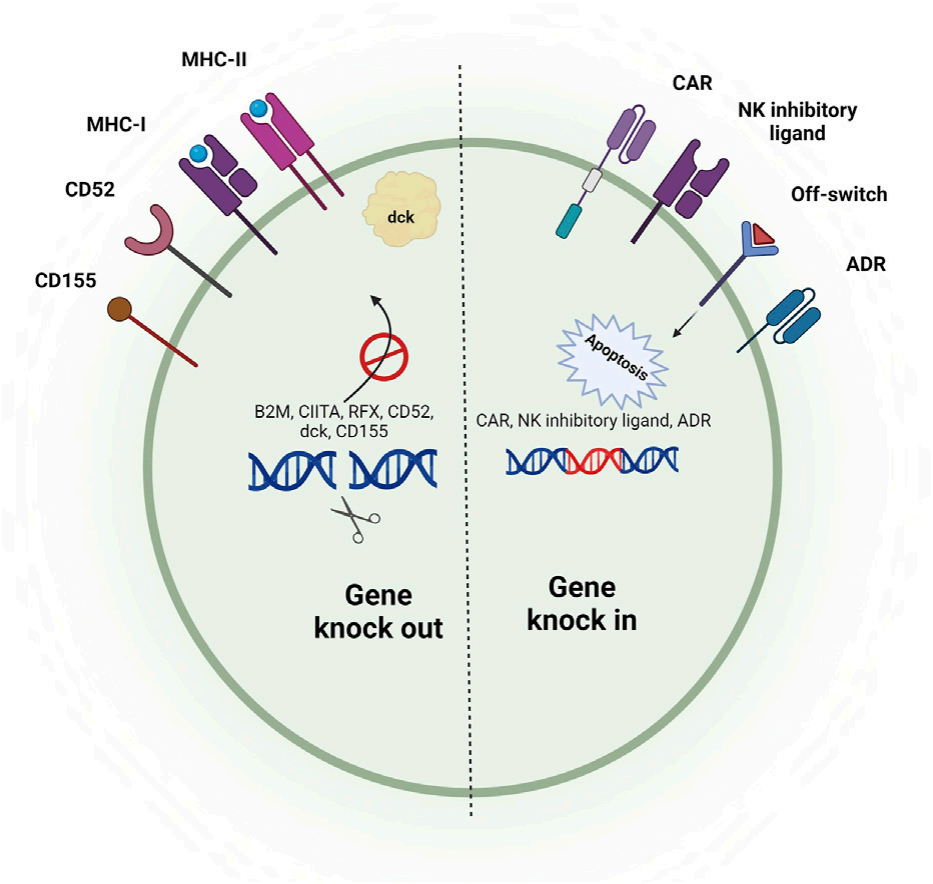
Stepwise process of differentiation of T-cells from iPSCs. To generate iT cells, iPSCs should first be differentiated into HSCs. By providing Notch signaling, HSCs can be differentiated toward functional iT cells. Premature TCR signaling (if a T cell clone is reprogrammed to IPSC) and/or CAR signaling can lead to generating innate-like T cells with CD8αα homodimer. Preventing pre-mature TCR/CAR signaling as well as providing stronger Notch signaling results in the generation of single-positive T-cells resembling peripheral blood-derived T cells.

Initial studies on generating T cells from iPSCs have shown that iPSC differentiation leads to iT cells with an innate-like phenotype that differs from conventional PB-derived T cells. These iT cells possess CD8αα and innate lymphocyte-related markers such as CD56 while lacking the expression of CD8αβ dimer, CD2, CD5, and CD28 (Themeli et al., 2013; Nishimura et al., 2013; Vizcardo et al., 2013; Ando et al., 2015). It has been shown that depleting double-negative T cells from mixed cultures before stimulation with an anti-CD3 monoclonal antibody enhances the formation of CD8αβ single-positive T cells because double-negative T cells can kill the double-positive T cells and impair their skew toward CD8αβ single-positive T cells (Maeda et al., 2016). On the other hand, it has been revealed that premature expression of TCR or early expression and constitutive signaling of CAR can prevent the formation of double-positive T cells and skew differentiation of iT cells toward innate/γδ-T-like CD8αα single-positive T cells (Figure 2) (van der Stegen et al., 2022). When T cells are used as the initial source for the generation of iPSCs (T-iPSCs), the expression of TCR occurs earlier than usual due to the pre-rearranged TCR. Signaling of pre-mature TCR prevents the transition to the double-positive stage, leading to the formation of an innate-like phenotype (Nianias and Themeli, 2019). In this regard, the use of OP9-DLL4 cells or a 3D thymic culture system instead of OP9-DLL1 cells can enhance the formation of double-positive T cells by providing stronger Notch signaling (van der Stegen et al., 2022; Vizcardo et al., 2018). Nonetheless, the expression of CAR transgene during the *in vitro* differentiation can reduce the expression level of Noth1, Notch3, and their downstream targets, leading to impairing the transition to the double-positive stage, even in the presence of DLL4-induced Notch signaling (van der Stegen et al., 2022). In addition to the downregulation of Notch signaling, early expression and constitutive signaling of CAR prevent the expression of PTCRA (Pre-T Cell Antigen Receptor Alpha), which has an important role in the development of αβ-T cells but not γδ-T cells (van der Stegen et al., 2022). Optimizing the CAR structure and regulating its expression time can avoid such a problem. Insertion of a 4-1BB co-stimulatory domain in place of a CD28 domain in the CAR construct has been shown to increase the transition to the double-positive stage and further formation of CD8αβ single-positive CAR-T cells (Li S. et al., 2023; Ueda et al., 2023). It has been revealed that the targeted insertion of a CAR construct with inactivated second and third ITAM to the TRAC locus can prevent TCR expression and constitutive signaling of CAR, leading to the restored Notch signaling, PTCRA expression, and enhanced formation of double-positive T cells. In the absence of TCR signaling, providing CAR target antigen and 4-1BB ligand effectively stimulates double-positive T cells and differentiates them toward CD8αβ single positive CAR-T cells (van der Stegen et al., 2022).

Differentiation of iPSCs toward the T lineage usually gives rise to the formation of CD8 single-positive T cells but not CD4 helper T cells, even when CD4^+^ T cells are used as the starting cellular material for the generation of iPSCs (Furukawa et al., 2023a). Although CD8^+^ CAR-T cells are considered the leading player of tumor killing, several studies have shown that the presence of both CD4^+^ and CD8^+^ CAR-T cells with a defined ratio within the final product can result in a better and more durable anti-tumor function (Sommermeyer et al., 2016; Turtle et al., 2016; Boulch et al., 2023; Lee et al., 2023; Melenhorst et al., 2022). While some recent studies have reported the successful generation of CD4^+^ iPSC-derived T cells using artificial thymic organoids (Montel-Hagen et al., 2019; Seet et al., 2017; Wang et al., 2022), the generation of these cells using feeder-free approaches remains challenging. Recently, Fong et al. developed a new feeder-free and serum-free method for the generation of CD4^+^ T cells from human iPSCs. In this method, stimulation of iPSC-derived CD4^+^ CD8^+^ double-positive T cells with PMA/Ionomycin skew their differentiation toward CD4 single-positive T cells (Fong et al., 2023).

## 4 Alternatives to iCAR αβ-T cells

Although αβ-T cells stand as the foremost cell type employed in CAR-based immunotherapy, the unknown and diverse repertoire of allogeneic CAR αβ-T cells comes with the risk of lethal GvHD. This issue necessitates gene editing steps to disrupt the expression of endogenous TCR. However, it should be noted that there is controversy regarding the role of endogenous TCR in the function of CAR-T cells (Smirnov et al., 2021). While the disruption of endogenous TCR has been successfully performed in various allogeneic CAR-T cell studies to prevent GvHD, the results of other studies indicate that the absence of endogenous TCR signaling impairs the persistence and function of CAR-T cells (Yang et al., 2017; Wang Z. et al., 2021).

**FIGURE 3:**
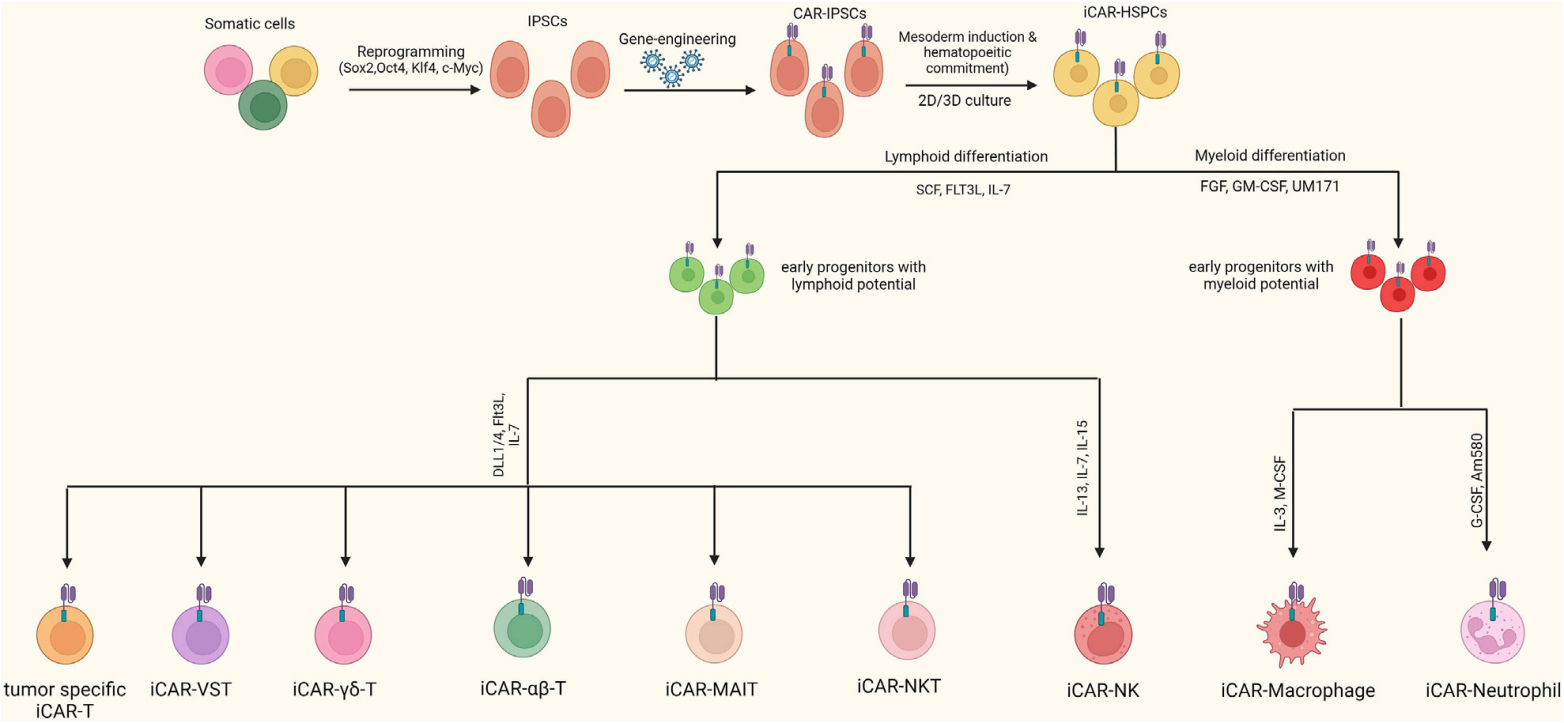
Generation of Various CAR-iPSC-derived cells. After engineering iPSCs with CAR, they can be differentiated toward CAR-T, CAR-NK, CAR-neutrophils, and CAR-macrophages. When a T-cell is used to generate iPSCs, the resulting cells express a TCR with an equivalent repertoire to that of the initial T-cell. In this regard, using T-cell clones with a defined TCR diversity as the starting material, final iCAR-T cells with specific TCRs that have a lower risk of GvHD can be generated.

In this regard, the use of iCAR T cells with a defined TCR repertoire, which lacks the risk of GvHD, or the use of other immune cells lacking TCR expression, can be used as alternatives to prevent GvHD. In recent years, several alternatives to CAR αβ-T cells have been introduced, including T cells with limited TCR diversity (gamma delta T, MAIT, iNKT, double-negative T, Virus-specific T, and tumor-specific T cells), cytokine-induced killer cells, natural killer cells, macrophages, and neutrophils (Figure 3). In addition to reducing the risk of GvHD, in some cases, these alternatives can promote anti-tumor function by providing both CAR-dependent and CAR-independent responses and prevent post-transplantation infections (Tang and Zhang, 2024). Nevertheless, the main obstacle in the front of clinical applications of these alternatives is the difficulty of isolating an adequate number of them from peripheral blood. In this regard, the use of an unlimited source such as iPSCs could overcome this limitation. Most of these alternatives have been successfully generated using iPSCs in recent years. The following paragraphs discuss the merits and drawbacks of these cells over the conventional iCAR αβ-T cells.

### 4.1 iCAR-T cells with low diverse/antigen-specific TCR

In 2013, for the first time, it was demonstrated that when a T cell clone is used to generate iPSC (T-iPSC), the resulting T cells from T-iPSC differentiation express a TCR with a repertoire similar to that of the initial T cell clone (Nishimura et al., 2013; Vizcardo et al., 2013). In this regard, T cell clones with a defined TCR repertoire can be used to generate iPSC to reduce the risk of GvHD. Although T-iPSC-derived T cells mainly express a TCR similar to the original T cell, in some cases, it has been reported that additional activity of recombinases in the double-positive stage can induce rearrangement of the TCR α chain and change its antigen specificity. Disruption of recombination activating gene 2 (RAG2) can prevent rearrangement of the TCR α chain and preserve the antigen specificity of T-iPSC-derived T cells (Minagawa et al., 2018). Reprogramming of T cell clones to iPSCs and further differentiation of iPSCs results in the generation of iT cells with telomers longer than the original clones. Due to the long telomeres, the fold expansion rate of iT cells is 5–50 times higher than that of the same clones derived from peripheral blood (Ando and Nakauchi, 2017). It should be noted that peripheral blood antigen-specific T cells are mainly terminally differentiated cells with short telomeres and are prone to die during reprogramming. It has been demonstrated that adding two factors, including NANOG and LIN28, to the classical Yamanaka factors can prevent apoptosis and increase the reprogramming efficiency (Netsrithong and Wattanapanitch, 2021). iCAR-T cells with low diverse/antigen-specific TCR and their generation from iPSCs are discussed in this section.

#### 4.1.1 Tumor-specific iCAR-T cells

If a T cell clone expressing a TCR against the tumoral antigen is used as the starting cellular material for generating iPSC, the endogenous TCR of the final iCAR-T cells shows similar antigen specificity. Thus, these iCAR-T cells can recognize and kill malignant cells via both CAR-directed and TCR-directed mechanisms without inducing GvHD. Several studies have used this method to manufacture tumor-specific iT cells. In these studies, leukemia WT1-, melanoma MART1-, EBV-induced lymphoma LMP2-, multiple myeloma BCMA-, and hepatocellular/lung carcinoma GPC3-reactive T cells have been successfully used for generating iPSCs and further redifferentiation to functional CD8^+^ iT cells with similar TCR specificity (Maeda et al., 2016; Vizcardo et al., 2013; Minagawa et al., 2018; Bae et al., 2024). Equipping these tumor-specific iT cells with CAR gives them a dual anti-tumor activity via both TCR and CAR.

#### 4.1.2 Virus-specific iCAR-T cells

The first documented application of virus-specific T cells in adoptive cell therapy was in the 1990s when it was shown that infusion of donor-derived T cells with specificity against viral antigens could prevent post-transplant infections in individuals who underwent HSC transplantation without inducing GvHD (Walter et al., 1995; Leen et al., 2013; Monzavi et al., 2021). Utilizing CAR-equipped allogeneic virus-specific T cells presents a promising approach to advance the development of an off-the-shelf product. The CAR of these cells can direct anti-tumor cytotoxic responses, while their TCR prevents post-transplant infections (a leading complication after CAR-T cell therapy). In recent years, virus-specific T-cell clones have been successfully equipped with various CARs and have shown promising safety and efficacy in several *in vitro/in vivo* assessments (Pule et al., 2008; Quach et al., 2024; Wang X. et al., 2021; Quach et al., 2022). It has also been demonstrated that recognizing viral antigens by endogenous TCR not only does not compromise the CAR-directed responses but also increases the duration of anti-tumor responses by boosting the *in vivo* proliferation of CAR-T cells (Wang X. et al., 2021; Cruz et al., 2013; Louis et al., 2011). In a clinical trial of CAR EBV-specific T cells in neuroblastoma patients, it has been demonstrated that these cells can induce a more durable anti-tumor response than their conventional counterparts, which is attributed to the costimulatory signals of their endogenous TCR (Pule et al., 2008). In addition, three other phase I clinical trials have been designed to investigate the safety and efficacy of virus-specific CAR-T cells (NCT00085930, NCT00840853, and NCT00840853).

Whereas virus-specific T cell clones can be efficiently isolated from peripheral blood by peptide-HLA multimers or cytokine capture techniques (Chang, 2021; Lambert et al., 2023), cells obtained from a single donor can only be sufficient for a single recipient. On the other hand, overexpansion of these cells to reach more of them can exhaust them (Quach et al., 2023). To overcome this issue, virus-specific T cell clones can be reprogrammed to T-iPSC and redifferentiated into T cells with the same TCR specificity. In this manner, an unlimited number of virus-specific T cells can be generated at clinical and industrial scales. Several studies indicate the feasibility of generating functional iPSC-derived virus-specific T cells using this method (Nishimura et al., 2013; Ando et al., 2015; Furukawa et al., 2023b; Haque et al., 2021; Honda et al., 2020). According to the promising safety and efficacy of allogeneic PB-derived CAR-modified virus-specific T cells, sourcing these cells from CAR-iPSCs could be a reliable strategy to provide an off-the-shelf product in a clinically relevant number.

#### 4.1.3 Gamma delta iCAR-T cells

Gamma delta (γδ) T cells are a subset of T cells that constitute less than 5% of peripheral blood lymphocytes. These cells express an HLA-unrestricted TCR in which one of the δ1, 2, 3, 4, and 5 chains are paired with one of the seven distinct varieties of γ chains. Based on the paired δ and γ chains, γδ-T cells can recognize CD1d- or MICA/B-presented lipid antigens or butyrophilin 3A1/2-presented phosphoantigens without requiring their processing with classical HLA systems. This unique TCR structure allows them to be utilized in third-party settings without the risk of inducing GvHD (Hu et al., 2023). The safety of γδ-T cells in allogeneic settings has been demonstrated in several preclinical and clinical studies (Hu et al., 2023; Ma et al., 2023). Due to their high cytotoxicity and low risk of GvHD, in recent years, γδ-T cells have emerged as a new candidate for developing an off-the-shelf CAR-modified cell product. CAR- γδ-T cells can suppress tumor cells via CAR-directed or CAR-independent responses (Rozenbaum et al., 2020). The latest is mediated by CD16-directed antibody-dependent cell cytotoxicity (ADCC) and natural killer (NK) cell cytotoxicity receptors (NKG2D, NKp30, NKp44, and NKp46) (Capsomidis et al., 2018; Dong et al., 2022). Moreover, having natural killer cell receptors makes them capable of preventing post-transplant infections (Chen et al., 2021).

*Ex vivo* expansion of γδ-T cells is mediated in the presence of zoledronate. Nonetheless, it has been revealed that zoledronate-mediated expansion of γδ-T cells is disparate between different donors, and γδ-T cells of some donors do not expand in response to the zoledronate (Wallet et al., 2021). In this regard, iPSCs could be reliable sources for generating high amounts of CAR γδ-T cells.

In recent years, some research groups have successfully generated iγδ-T cells using γδT-iPSCs (Wallet et al., 2021; Watanabe et al., 2018; Murai et al., 2023; Zeng et al., 2019; Jeon et al., 2023). In 2018, Watanabe et al. developed a new method for the generation of γδT‐iPSCs without requiring the sorting of peripheral blood γδ T cells. In this method, peripheral blood mononuclear cells are cultured in the presence of IL-2 and zoledronate, leading to the specific activation and enrichment of γδ-T cell clones. Thus, due to the dominancy of γδ T cells, most of the reprogrammed cells are γδT-iPSCs which can be redifferentiated to iγδ-T cells (Watanabe et al., 2018). In 2019, Zeng et al. used an NK-promoting protocol to redifferentiate γδT‐iPSCs into iγδ T cells, which led to the generation of iγδ T cells with the expression of Natural killer receptors. These cells, which were different from natural peripheral blood γδ-T cells, were called “iγδ NKT cells” (Zeng et al., 2019). In newly published research, Murai et al. redifferentiated γδT‐iPSCs toward γδ-T cells. They revealed that the resulting cells are similar to a minor subtype of peripheral blood γδ-T cells. In contrast to the majority of the PB-derived γδ-T cells, the generated iγδ-T cells had a low expression level of CD2 and CD5, low antigen-presenting ability, and lower interferon gamma-secretion activity while a higher expression level of NK cell markers, KIT, and CD7 (Murai et al., 2023). These results indicate that iγδ-T cells are not completely on par with natural PB-derived γδ-T cells. Thus, subsequent studies are needed to completely elucidate the molecular and functional similarities and differences between iγδ-T cells and PB-derived γδ-T cells. In recent years, significant progress has been made in developing iγδ-T cells using CGMP-compliant, feeder-free, and serum-free protocols, which can facilitate the translation of these cells from bench to beside (Wallet et al., 2021; Jeon et al., 2023).

There are also promising pioneering data regarding iCAR γδ-T cells. Scientists in “Century Therapeutics” recently generated iCAR γδ-T cells from γδT‐iPSCs in a feeder-free condition. These cells showed favorable anti-tumor responses in the preclinical assessments (Wallet et al., 2021). Quinn et al. revealed that iCAR- γδ T cells mediate anti-tumor activity via CAR-dependent cytotoxicity and ADCC. To sustain the *in vivo* durability of iCAR γδ-T cells, they engineered these cells to express a membrane-bound IL-15 (Quinn et al., 2022).

#### 4.1.4 iCAR iNKT cells

Invariant natural killer T cells (iNKT) cells constitute less than 1% of PB T lymphocytes and are characterized by the expression of both T and NK cell markers. These cells pose a semi-diverse αβ-TCR, which are HLA-unrestricted and recognize glycolipid antigens presented by CD1d (Look et al., 2023). Thus, they are potential candidates for adoptive cell therapy in third-party settings without the risk of developing GvHD. On the other hand, these cells are very cytotoxic, which makes them an attractive cell type that can be modified with CAR to kill malignant cells (Hadiloo et al., 2023a). The remarkable safety and effectiveness of CAR iNKT cells have been demonstrated in a variety of preclinical assessments (Liu et al., 2022b). It has been revealed that in some cases, CAR iNKT cells have advantages over the conventional CAR αβ-T cells. Their CD1d-restricted TCR can synergize with CAR to kill CD1d-positive tumors. Their NK receptors can also sustain anti-tumor responses and prevent CAR-target antigen-negative relapses. They pose a broad spectrum of chemokine receptors, facilitating their infiltration into tumor sites (Rotolo et al., 2018). In a preclinical assessment, it has been shown that these cells can pass the blood-brain barrier and suppress the secondary brain lymphoma more efficiently than their CAR αβ-T counterparts (Rotolo et al., 2018). Moreover, these cells can reduce the immunosuppressive effects of the tumor microenvironment by suppressing myeloid-derived suppressor cells and tumor-associated macrophages (Karadimitris et al., 2019). Currently, there are three ongoing phase I clinical trials of CAR iNKT cells (NCT03774654, NCT04814004, NCT03294954). Published results indicate the favorable anti-tumor activity of CAR iNKT cells with a high safety profile (Heczey et al., 2020).

*Ex vivo* activation and expansion of CAR iNKT cells can be mediated using the administration of α-galactosylceramide (α-GalCer). The very limited number of iNKT cells in peripheral blood necessitates long-term *ex vivo* cultivation to reach a clinically valuable number of these cells. Nonetheless, chronic exposure of iNKT cells to α-GalCer may induce their exhaustion, anergy, or death or differentiate them toward anti-inflammatory T helper 2 and regulatory T phenotypes (Hadiloo et al., 2023a). The use of iPSCs as the source of iNKT cells can overcome this limitation and provide iNKT cells in a large number for industrial applications. First time in 2010, Watarai et al. generated iPSC-derived iNKT cells. They reprogrammed mouse splenic iNKT cells to iPSCs and redifferentiated them into functional iNKT cells (Watarai et al., 2010). In 2016, Yamada et al. developed human Vα24 + iNKT cell-derived iPSCs and successfully redifferentated them into iPSC-derived iNKT cells. They showed that these cells have reliable anti-tumor activity in animal models even better than PB-derived iNKT cells (Yamada et al., 2016). The feasibility of the generation and anti-tumor function of iPSC-derived iNKT cells has also been demonstrated by other preclinical studies (Kitayama et al., 2016; Urakami et al., 2022). iPSC-derived iNKT cells are currently being evaluated in a phase I clinical trial for patients with advanced head and neck cancer (HNC) (jRCT2033200116).

Although iPSC technology has not yet been applied for the generation of CAR-iNKT cells, given the successful generation of iNKT cells from iPSCs (Watarai et al., 2010; Yamada et al., 2016; Kitayama et al., 2016; Urakami et al., 2022; Aoki et al., 2023) and the encouraging results of PB-derived CAR-iNKT cells (Ponnusamy et al., 2023; Rowan et al., 2023; Ren et al., 2023; O’Neal et al., 2023), it is expected that the generation of iPSC-derived CAR-iNKT cells will be carried out in future studies.

#### 4.1.5 iCAR MAIT cells

Mucosal-associated invariant T (MAIT) cells are an innate-like T cell subtype expressing an HLA-unrestricted semi-invariant TCR. They recognize metabolites of vitamin B2 presented by MR1 and have a crucial role in response to vitamin B2-producing microbial agents (Hinks and Zhang, 2020). Due to the HLA-unrestricted mechanism of action and the lack of production of riboflavin derivatives by human cells, MAIT cells have the potential to be utilized in third-party settings without the risk of developing GvHD (Talvard-Balland et al., 2024). In recent years, several preclinical studies have been conducted to evaluate CAR-MAIT cells, and their results indicate the safety and efficacy of these cells (Dogan et al., 2022; Bohineust et al., 2021; Li YR. et al., 2022). In addition to CAR-directed responses, CAR-MAIT cells can be activated in CAR-independent manners comprising TCR-MR1 interaction (in MR1-positive tumors), NK receptors (such as NKG2D), and various cytokines (IL-2, -15, and −18) (Li YR. et al., 2022; Hinks and Zhang, 2020). By killing the tumor-associated macrophages via CAR-independent responses, CAR-MAIT cells can also reduce the immunosuppressive effect of the tumor microenvironment (Li YR. et al., 2022). From the safety prospects, due to the lower secretion of proinflammatory cytokines such as IFN-γ, compared to their CAR αβ-T counterparts, CAR-MAIT cells are associated with a lower risk of CRS (Dogan et al., 2022).

While some of most cells have a T helper 2 (Th2) phenotype and tumor-promoting function, the majority of them are highly cytotoxic and are able to exert potent anti-tumor responses via their FASL, TRAIL, and the secretion of granzyme A, B, K, H, and M, and perforin. It has also been shown that equipping MAIT cells with CAR can direct their polarization toward the anti-cancer phenotype (Li YR. et al., 2023). Whereas in mucosa-rich tissues, the abundance of MAIT cells reaches up to 40% of all T cells, in peripheral blood, they constitute 1%–10% of all T cells. MAIT cell’s *ex vivo* expansion is conducted by sorting them from PBMCs and subsequent culture of them in the presence of MR1 Tetramer-Based artificial antigen-presenting cells or irradiated mononuclear cells that are loaded with 5-OP-RU. Nonetheless, using this method, the expansion rate of MAIT cells lies between 60 and 200-fold (Li YR. et al., 2023). Therefore, generating a clinically valuable number of CAR-MAIT cells can be considered as one of the main limitations of their clinical translation. This underscores the need to provide a source such as iPSCs to generate MAIT cells in a clinically meaningful number. On the other hand, given the paucity of MAIT cells in the peripheral blood of mice, iPSC-derived MAIT cells can be used in preclinical studies to understand better the features of MAIT cells and their roles in diseases (Sugimoto et al., 2024).

In 2013, Wakao et al. conducted a pioneering study. They reprogrammed cord blood-derived MAIT cells into iPSCs, redifferentiated them toward MAIT cells, and called them m-reMAIT cells. Although the generated m-reMAIT cells were different from PB-MAIT cells in terms of differentiating state, homing chemokine receptors, and surface markers, they were capable of migrating to various organs and preventing the occurrence of *Mycobacterium* abscessus in immunocompromised mice (Wakao et al., 2013). In 2022, in another study, Wakao and colleagues revealed that m-reMAIT cells can efficiently migrate to various organs. They showed that injection of m-reMAIT cells before inoculation of Lewis lung carcinoma (LLC) increases the survival of mouse models, while injection of these cells after LLC inoculation does not suppress tumor growth (Sugimoto et al., 2022).

Despite the abovementioned items, there is very little data about m-reMAIT cells. Future studies should be conducted to completely elucidate the difference between m-reMAIT cells and PB-derived MAIT cells. It is unclear if these cells have a migration and anti-tumor activity on par with their PB-derived counterparts. It should also be assessed whether modifying these cells with CAR can improve their cytotoxicity or not.

### 4.2 iCAR NK cells

NK cells are the second most widely utilized cell type in CAR-directed target therapy. CAR-NK cells are being evaluated in more than 50 registered clinical trials (Zhang et al., 2023). From both safety and efficacy perspectives, they have several properties that propose them as a promising cell type for modification with CAR. NK cells are parts of the innate immune system and do not express TCR, allowing their use in allogeneic settings without the risk of GvHD (Hadjis and McCurdy, 2024). CAR-NK cells have a potent cytotoxic activity which can be exerted upon CAR signaling or CAR-independent mechanisms, including ADCC and various activating receptors (NKp44/46/80, NKG2D, KIR2DS, DNAM1, 2B4, and NTBA). These CAR-independent mechanisms of action make them more efficient than CAR-T cells in eradicating tumor clones that are negative for CAR target antigen and preventing target antigen-negative relapses (Moradi et al., 2023; Zhang et al., 2024). In contrast to CAR-T cells, which can develop life-threatening CRS by secreting IL-1/6 and TNF-α, CAR-NK cells secrete a different set of cytokines, such as GM-CSF, IL-3, and IFN-γ, which come with a lower risk of CRS (Zhang X. et al., 2022). Because of the lower adverse events, CAR-NK cell therapy has the potential to be performed in outpatient settings (Zhong and Liu, 2024).

Peripheral blood (PB), cord blood (CB), and NK-92 cell line are three classical sources of NK cells. Although PB-NK cells are highly cytotoxic, leveraging them in CAR-NK cell therapy has specific challenges. Due to their small number in PB (about 10% of PB-lymphocytes) and low expansion capacity, their long-term *ex vivo* cultivation is necessary to attain a clinically valuable quantity. On the other hand, prolonged expansion of NK cells within culture conditions can adversely affect their viability and cytotoxicity (Yang et al., 2020; Lamers-Kok et al., 2022; Heipertz et al., 2021). In contrast, cord blood contains more NK cells (about 30% of its lymphocytes) with higher expansion capacity than PB-NK cells. Moreover, global and national cord blood banks worldwide make it easy to access CB-NK cells (Zhang et al., 2024). Nonetheless, the low cytotoxicity of CB-NK cells due to their immature phenotype is their main bottleneck (Sarvaria et al., 2017). Moreover, due to the low transduction/transfection rate, genetic manipulation of PB- and CB-NK cells is challenging (Schmidt et al., 2020). NK cells have a relatively short lifespan in the body (less than 2 weeks), which may necessitate multiple steps of NK transfusion (Pan et al., 2022), underscoring the need for their large-scale manufacturing. The only approved NK cell line for human applications is NK-92. Nonetheless, due to the worries about developing secondary malignancies (due to their cancerous nature), these cells should be irradiated before administration, significantly reducing their lifespan. Additionally, without IL-2, these cells will die within 3 days; however, systemic administration of IL-2 comes with the risk of serious adverse events. Finally, due to the lack of CD16 expression, NK-92 cells cannot mediate ADCC (Zhang et al., 2019; Klingemann, 2023).

iPSC technology can provide a reliable source for the continuous production of NK cells with homogeneous phenotypes. Various desired genetic manipulations can be performed in the iPSC stage, and the iPSC clone harboring the intended changes can be selected and differentiated toward functional CAR-NK cells. These genetic alterations can be made to increase the persistence, cytotoxicity, and tumor infiltration of CAR-NK cells, as well as reduce their immunogenicity (Goldenson and Kaufman, 2021). Since the pioneering work of differentiation of NK cells from ESCs (Woll et al., 2005). Several steps have been taken to generate iPSC-derived NK cells. Initial studies have demonstrated the feasibility of manufacturing functional iPSC-NK using classical differentiation methods, including co-culture with stromal cells, matrix-coated Petri dishes, and spin embryoid body (Knorr et al., 2013; Goldenson et al., 2022). Nonetheless, cGMP-compliant production of NK cells on an industrial scale remains challenging. Feng et al. recently registered a patent to develop a 3D bioreactor platform to generate homogeneous iPSC-NK cells on an industrial-scale and serum-free cGMP-compliant condition. In this system, the release of iNK cells starts on the 15th day and continues until the 45th day. Over 95% of released cells are positive for CD56, NKG2D, NKP44, and NKP46 and negative for CD3, TCR, and B-cell markers. The final yield of this system is about 10^10^ NK cells/300 mL (Qiang et al., 2020).

It has been revealed that iNK cells are very similar to primary NK cells. However, some differences can exist between iNK and primary NK cells. For example, Zeng et al. have shown that when PB-derived cells are reprogrammed to iPSC, the generated iNK cells do not express inhibitory killer cell immunoglobulin-like receptors (KIRs) which makes them more functional than primary NK cells (Zeng et al., 2017). CD16a, the responsible molecule for mediating ADCC, has allelic variation binding affinity to the Fc region of IgG. Moreover, metalloprotease ADAM17 cleaves the CD16a from the surface of activated NK cells. Inserting a non-cleavable high-affinity CD16 (hnCD16) or a CD64/16a chimeric receptor can increase the ADCC function of NK cells. by engineering iCAR-NK cells to express these Fc receptors, CAR-NK cell therapy can be combined with monoclonal antibodies to eradicate tumor cells via both CAR-directed mechanisms and ADCC (Dixon et al., 2020; Zhu et al., 2020). This strategy is currently being evaluated in clinical trials (discussed in the subsequent sections).

### 4.3 iCAR macrophages

Macrophages are tissue-resident parts of the innate immune system that are distributed throughout the body and play a vital role in defense against exogenous and endogenous harmful agents and in maintaining tissue hemostasis (Na et al., 2023). Macrophages are mainly classified into two distinct subsets: M1 macrophages and M2 macrophages. These two subsets of macrophages are phenotypically and functionally different. M1 macrophages have anti-tumor activity and are characterized by the expression of CD68, 80, 86, and MHC-II, and M2 macrophages that promote tumor growth and are characterized by the expression of CD163, 204, 206, and secretion of immunosuppressive cytokines such as IL-10 and tumor growth factor-β (Hadiloo et al., 2023b; Jayasingam et al., 2019). In terms of tumor infiltration, macrophages outperform other types of immune cells. Research indicates that over half of tumor-infiltrating immune cells are macrophages exhibiting the M2 phenotype, commonly referred to as tumor-associated macrophages (TAMs) (Liu J. et al., 2022).

Several research groups have recently focused on developing CAR-modified macrophages as off-the-shelf CAR-based products. CAR macrophages have several advantages over other CAR-engineered cells. The lack of TCR expression allows them to be used in third-party settings without the risk of GvHD. The natural tendency of CAR-macrophages to the tumor sites makes them a better therapeutic candidate for solid and dense tumors. Moreover, their long-living activity in the tumor sites provides a sustainable anti-tumor response (Chen et al., 2024). It has been demonstrated that modifying the macrophages with CAR skews their polarization toward the M1 phenotype (Dong et al., 2023). Upon CAR signaling, these cells mediate direct anti-tumor activity by phagocytosis of malignant cells or indirectly promote anti-cancer immune responses by various mechanisms, including secretion of inflammatory compounds (such as TNF-α), antigen presentation to T cells, promoting the activity of antigen-presenting cells, and increasing permeability of tumor microenvironment to immune cells (Chen et al., 2024; Klichinsky et al., 2020). CAR-macrophages are capable of being loaded with the intended therapeutic cargos to deliver them directly to targeted tumor sites (Liu et al., 2022d; Yang S. et al., 2024).

Despite the merits of CAR macrophages, several bottlenecks limit the generation of these cells from natural sources. Genetic engineering of macrophages is difficult even by lentiviral/retroviral vectors (Gao et al., 2023). On the other hand, they are very rare in peripheral blood, making it impossible to reach many of them from peripheral blood to clinical use. To overcome this limitation monocytes can be isolated from peripheral blood, modified with CAR, and differentiated into CAR macrophages (Yang B. et al., 2024; Gabitova et al., 2022). Nonetheless, the paucity of monocytes within PB leukocytes and their limited expansion rate hinder the generation of CAR macrophages on an industrial scale. It has also been reported that monocyte-derived macrophages are not completely similar to tissue-resident macrophages (Shyam Sushama et al., 2022). To overcome these limitations, iPSCs can serve as reliable and potentially unlimited sources for generating macrophages. It has also been shown that compared to monocyte-derived macrophages, iMacrophages are better models for tissue-resident macrophages (Shyam Sushama et al., 2022).

Differentiating iMacrophages from iPSCs requires the regulation of a stepwise process with the timed addition of the required cytokines and growth factors. This process involves the differentiation of iPSCs to HSCs (It was described earlier), myeloid specification of HSCs, formation of CD14-positive monocyte-like precursors, and differentiation of these precursors toward functional iMacrophages (Figure 3) (Lyadova et al., 2021). In recent years, various research groups have successfully generated iMacrophages using different methods, including embryoid body-dependent, stromal cell line-dependent, and xeno-free methods (Klepikova et al., 2022; Yanagimachi et al., 2013). Results of these studies have shown that differentiation of iPSCs leads to the generation of iMacrophages with both M1-and M2-like phenotypes. However, phenotypical and functional biases exist between the iMacrophages generated by different methods. iMacrophages generated using stromal cells have been reported to be phenotypically and functionally biased toward M2 macrophages, whereas embryoid body-derived iMacrophages tend to have M1-like characteristics (Klepikova et al., 2022). However, several results indicate that iMacrophages are reliable cells that can be used instead of monocyte-derived macrophages for cell therapy (Lyadova and Vasiliev, 2022). Given that iMacrophages are terminally differentiated cells and cannot be expanded *in vitro*, it is crucial to generate them on large scales for clinical applications. In a recently published elegant study, Ackermann et al. developed a novel method to generate iMacrophages in a xeno-free and chemically defined intermediate-scale bioreactor platform. This method allows the continuous production of iMacrophages from 3D hematopoietic organoids. This method yields functional, reproducible, and highly pure iMacrophages that can be harvested weekly for multiple weeks (Ackermann et al., 2024).

The feasibility of generating iCAR macrophages and the efficiency of these cells against solid tumors and hematologic malignancies has been demonstrated in various preclinical studies (Kong et al., 2022; Zhang J. et al., 2022; Zhang et al., 2020; Shah et al., 2024; Shen et al., 2024). In a Proof-of-concept study, Abdin et al. successfully generated anti-CD19 iCAR macrophages with an M1-like phenotype and CAR-dependent activity. This study used an automated CERO 3D bioreactor to produce iCAR macrophages on an intermediate scale. The overall yield of this method was up to ∼5.73 × 107 cells/40 mL (Abdin et al., 2023). This method has the potential to translate into industrial-compliant scale.

### 4.4 iCAR neutrophils

Although CAR-neutrophils are considered a new type of CAR-engineered cells, the first report on the production of CAR neutrophils was in 1998 (Roberts et al., 1998). However, the Modification of neutrophils with CAR to fight cancer cells was not widely investigated until recent years. CAR neutrophils have several features that render them attractive alternatives to CAR-T cells. They are parts of the innate immune system and do not express TCR; thus, CAR-neutrophils can be safely utilized in third-party settings without the risk of triggering GvHD (Chang et al., 2023). They can efficiently penetrate physical barriers such as blood-brain barrier and blood-tumor barrier. This makes them superior to T cells in accessing dense solid tumors and brain malignancies (Chang et al., 2022). Recent studies indicate that CAR neutrophils are versatile tools that can be used as cytotoxic cells and/or delivery tools for carrying anti-cancer agents into tumor sites to avoid their systemic toxicity (Chang et al., 2023; López-Arredondo et al., 2024). While it has been shown that some neutrophils show N2 phenotype and exert tumor-promoting responses (similar to M2 macrophages), equipping neutrophils with CAR, skew their polarization toward anti-cancer N1 phenotype (Chang et al., 2023; Chang et al., 2022; Harris et al., 2023; Majumder et al., 2022). The anti-tumor function of CAR neutrophils is mediated in a CAR-directed antigen-specific manner and includes direct phagocytosis, NETosis, and the production of reactive oxygen species (ROS) (Liang et al., 2023).

Despite the abundance of neutrophils within the peripheral blood, two main bottlenecks hinder the clinical applications of CAR-neutrophils. First, the genetic engineering of neutrophils with the currently available gene engineering tools has low efficiency and leads to suboptimal expression of CAR by neutrophils. Second, neutrophils have a very short lifespan and are not expandable *ex vivo* or *in vivo,* underscoring the need for generating a large amount of them to support their repeated administration (Chang et al., 2022).

Due to the possibility of differentiation into all adult cell lineages, iPSC technology can provide a scalable source for generating CAR neutrophils. The feasibility of differentiation of neutrophils from iPSCs has been demonstrated in several studies. In 2019, Brok-Volchanskaya developed a serum-free and xeno-free method capable of generating functional neutrophils from iPSCs. In this method, iPSCs are first differentiated into hematoendothelial progenitors using ETV2-modified mRNA. In the presence of GM-CSF, FGF2, and UM171, these hematoendothelial progenitors can subsequently be directed to form CD34^+^ CD33^+^ myeloid progenitors. Using this method these myeloid progenitors can be harvested every 8–10 days for up to 1 month. The subsequent culture of the generated myeloid progenitors in the presence of G-CSF differentiates them into mature functional iNeutrophils. They state that this method can produce a considerable number of iNeutrophils within 14 days (Brok-Volchanskaya et al., 2019).

There is an increasing focus on the research surrounding the generation of CAR neutrophils from CAR-iPSCs. The published results indicate that CAR neutrophils can be successfully generated from iPSCs and can be used as antigen-specific cytolytic agents or drug-delivery vehicles (Chang et al., 2023; Chang et al., 2022; Harris et al., 2023; Majumder et al., 2022; Chang et al., 2023). It seems that the use of CRISPR/Cas9 for the targeted insertion of CAR within the safe genomic harbors such as AAV1 locus leads to better and consistent expression of CAR among the resulting iCAR-neutrophils (Chang et al., 2023; Chang et al., 2022; Harris et al., 2023)Nonetheless, these cells are new to the block of CAR-based immunotherapy and require further optimizations, such as developing a protocol for large-scale manufacturing of iCAR neutrophils and optimizing the CAR construct to elicit the optimal function of iCAR neutrophils. Further preclinical and clinical evaluations are also needed to validate the safety and efficacy of iCAR neutrophils.

## 5 Clinical translation of iCAR-T products: Challenges and considerations

### 5.1 Genomic instability

Genomic instability is a serious concern in iPSC-based therapeutics as this can lead to the malignant transformation of iPSC-derived cells and the development of secondary malignancies. During reprogramming, the metabolism of reprogrammed cells is shifted from oxidative respiration to oxidative glycolysis, which increases the risk of genetic changes by creating an oxidative stress condition within the cells (Steichen et al., 2019). The oncogenic effect of reprogramming factors (specifically c-Myc), prolonged *in vitro* culture, and enzymatic passaging are other factors that can promote the risk of genomic instability (Beers et al., 2012; Netsrithong et al., 2024). Analyzing the 20-metaphase karyotype using G-binding is the most frequently used method for detecting chromosomal instabilities. Nonetheless, this method analyzes less than 30 cells and is insensitive to small genetic changes. Whole genome sequencing, Single nucleotide polymorphism (SNP) arrays, and KaryoLite BoBs^®^ are newer and more precise techniques for screening the genomic alteration of iPSCs (Steichen et al., 2019; O'Shea et al., 2020). KaryoLite BoBs^®^ and SNP arrays analyze more than 10^6^ cells in a shorter period and at a lower cost than G-binding. Nonetheless, these techniques are not able to detect inversions/balanced rearrangements (O’Shea et al., 2020). Given the lack of a method with 100% sensitivity and specificity, it is recommended to combine various methods to increase accuracy. When a gene editing (specifically multiplex gene editing) strategy is applied, screening for detection of off-target and on-target genotoxicities is crucial. The methods for predicting or detecting these unintended events are comprehensively discussed in our previous paper (Moradi et al., 2024).

### 5.2 Immunogenicity

Due to the allogeneic properties of iCAR-T products, they are rapidly rejected by the host immune system. In this regard, gene editing strategies can be applied to reduce the immunogenicity of iCAR-T products (as comprehensively discussed in the previous sections). Another strategy to enhance the *in vivo* survival of injected cells is the use of high-dose chemotherapy to deplete host immune cells completely before the administration of iCAR-T cells (Moradi et al., 2023).

### 5.3 Tumorigenicity

Tumorigenicity is one of the leading concerns about the clinical use of iPSC-derived products. This can occur due to the existence of undifferentiated iPSCs within the final product or malignant transformation of intermediate or final cells (Patel et al., 2019). Despite the presence of highly precise quality control assessments, the evaluation of all the generated cells is not possible. The manufacturing process and in-process/finished product quality must be rigorous to reduce the risk of tumorigenesis. *In vitro* quality control assays should be coupled with *in vivo* functional assays. In these *in vivo* methods, produced cells are injected into immunodeficient mice, and their *in vivo* behavior is monitored for up to 1 year (Moradi et al., 2024).

Introducing reprogramming factors by non-integrating vectors is preferred to integrating methods. While the use of non-integrating vectors to introduce reprogramming factors into the cells reduces the reprogramming efficiency, the efficiency of some non-integrating vectors, specifically mRNA vectors, is sufficient as much as providing a suitable number of iPSCs (Kogut et al., 2018).

Finally, engineering iPSCs to generate iCAR-T cells expressing an off-switch receptor ensures that the transfused cells can be rapidly depleted from the recipient’s body in case of unintended adverse events (comprehensively discussed in section 3.3).

### 5.4 Required technical optimizations

There is a real-time need to optimize the entire process, including the enhancement of reprogramming efficiency, shortening the time of *in vitro* culture, and the use of cost-effective and chemically defined media. With a deeper understanding of the mechanisms involved in the iPSC differentiation process, this process can be controlled more and directed towards generating the desired cell. For example, optimizing iPSC differentiation to generate both CD4^+^ and CD8^+^ T cells can lead to better therapeutic efficiency (Furukawa et al., 2023a).

### 5.5 The matter of timeline, cost, and scale-up manufacturing

The production of iCAR-T cells is a costly and time-intensive process that can take several months. Since the prolonged *in vitro* culture increases the risk of contamination and genomic instability, reducing the manufacturing timeline while maintaining regulatory standards is crucial. Integrating different steps of iCAR-T cell manufacturing in a continuous, automated, and closed-loop system can significantly diminish the overall time and cost. Implementing a continuous system also provides the possibility of real-time monitoring of the manufacturing process and prompt rectification of the errors that occur, preventing production delays (Abou-El-Enein et al., 2021). Developing cGMP-compliant pre-optimized iPSC cell lines can dramatically reduce the production timeline by circumventing the initial steps, which include iPSC generation and quality control (Madrid et al., 2024). Another potential strategy to reduce the time and cost of manufacturing is the utilization of a split, universal, and programmable (SUPRA) CAR system. In this strategy, iPSCs are engineered to express a constant signal transduction receptor called zipCAR which lacks the antigen binding domain. After the differentiation of zipCAR-expressing iT cells from IPSCs, the addition of antigen binding domain (zipFv) leads to the formation of functional CAR capable of antigen recognition and signal transduction. Using this strategy various iCAR-T cells with different antigen specificities can be differentiated from the same iPSC line (Zhao et al., 2018). Engagement with regulatory authorities from the initial stages and throughout the development process can facilitate clinical translation by reducing the required time for regulatory review. A platform-based approval strategy in which every manufacturing stage (e.g., reprogramming or gene editing) is standardized and approved separately can considerably cut costs and manufacturing time since each standardized production step can also be applied to subsequent products using this strategy (Ammar et al., 2023).

Scaling and closing the current methods for generating iCAR-T cells is crucial to translating these therapeutics into clinical settings. The production of iCAR-T cells in cGMP-compliant conditions and on an intermediate scale is feasible using currently available methods. However, the yields of these methods can only support an initial phase safety assessment clinical trial (Iriguchi et al., 2021). To bring these products to the market, it is necessary to scale up the manufacturing of iT cells using larger equipment like automated stirred-tank bioreactors. Recently, Trotman-Grant et al. developed and patented a scalable serum-free feeder-free method for differentiating a clinically relevant number of T cells from stem cells. In this method, microbeads of a specific size are coated with DL4 and induce the commitment of T cells with the cooperation of lymphoid factors in suspension culture conditions (Trotman-Grant et al., 2021; Juan Carlos et al., 2019).

## 6 Clinical trials of CAR-iPSC-derived products

The promising results of iPSC-derived cells in preclinical have opened the door for their assessment in clinical settings. According to the Clinical study database for human pluripotent stem cell (hPSC)-based cell therapies until the first of July, 153 clinical trials have been registered to evaluate hPSCs or their derivatives (Guhr et al., 2024). Several companies worldwide are also working on developing iPSC-derived CAR-modified cell-based products. Some of these groundbreaking products have advanced to clinical trials and are currently undergoing evaluation in phase I trials (Table 2).

**TABLE 2:** Clinical trials of iPSC-derived CAR-modified cells.

Cell type	Product name	NCT number	Developer	CAR target Ag	Genome modifications		Disease	Study phase
					Inserted genes	Disrupted genes		
iCAR-T	FT819	NCT04629729	Fate Therapeutics	CD19	CD19	TRAC	CD19^+^ B-cell malignancies	I
	FT825	NCT06241456	Fate Therapeutics	HER2	HER2 CAR, IL7RF, hnCD16a, CXCR2, TGFβ-signal redirection receptor	TRAC, CD38	Advanced solid tumors	I
iCAR-NK	FT522	NCT05950334	Fate Therapeutics	CD19	CD19 CAR, hnCD16, IL-15/IL-15Rα, anti-4-1BB ADR	CD38	CD19^+^ B-cell malignancies	I
	FT576	NCT05182073	Fate Therapeutics	BCMA	BCMA CAR, hnCD16, IL-15/IL-15Rα	CD38	Multiple myeloma	I
	FT596	NCT04555811	Fate Therapeutics	CD19	CD19 CAR, hnCD16, IL-15/IL-15Rα	none	CD19^+^ B-cell malignancies	I
	CNTY-101	NCT05336409/NCT06255028	Century Therapeutics	CD19	CD19 CAR, IL-15, Safety switch, HLA-E	β2M and CIITA	CD19^+^ B-cell malignancies/lupus Erythematosus	I
	Anti-CD19 iCAR NK Cells	NCT03824951	Allife Medical Science and Technology Co., Ltd	CD19	Undisclosed	Undisclosed	CD19^+^ B Cell Lymphoma	I
	CLL1 CAR-NK cell	NCT06027853	Zhejiang University	CLL1	Undisclosed	Undisclosed	AML	I
	iPSC-NK cells	NCT06367673	Zhejiang University	CLL1 or CD33	Undisclosed	Undisclosed	AML	I

“Fate Therapeutics” is an outstanding company focused on developing off-the-shelf iCAR-T, iCAR-NK, iT, and iNK products for various therapeutic purposes. Several of this company’s iCAR-T and iCAR-NK products are currently undergoing assessment in phase I clinical trials. The ICAR-T pipeline of Fate Therapeutics comprises FT819 (anti-CD19 iCAR-T), FT825/ONO-8250 (anti-HER2 iCAR-T), and FT836 (anti-MICA/B ICAR-T), except for the last one two others have been entered clinical trials. FT819 is manufactured using CRISPR/Cas9 for site-directional insertion of a novel 1xx CD19 CAR into the TRAC locus. This strategy has several advantages, including prevention of GvHD (due to the disruption of endogenous TCR), prevention of insertional mutagenesis (due to the site directional insertion of CAR), and prevention of skewing T cell progenitors toward CD8αα+ innate-like T cells (due to the lacking the expression of premature TCR and prevention of CAR tonic signaling during the differentiation). Fate Therapeutic’s stage-specific differentiation protocol generates a homogenous population of iCAR-T cells, which are completely negative for TCR, and 99% of them express CAR (Yuan et al., 2021). The preclinical results indicate that FT819 is safe and can control tumor growth as effectively as primary CAR-T cells (Clarke et al., 2018; Chang et al., 2019; Mandal et al., 2020). FT819 is currently under evaluation in a Phase I dose-finding study (NCT04629729) as a monotherapy (single dose of 90/180 million cells or three doses of 30 million cells) or in combination with IL-2 (single dose of 90/180 million cells) for 12 patients with CD19^+^ B-cell malignancies. Before product administration, all patients received fludarabine (30 mg/m2) and cyclophosphamide (500 mg/m2) for three consecutive days. The interim results of this ongoing trial have recently been released, indicating this product’s promising safety and efficacy. There were no reports of GvHD, ICANS, dose-limiting toxicity, Grade ≥3 CRS, or other treatment-related Grade ≥3 serious adverse events among the 15 subjects treated with FT819. Of 15 efficacy-evaluable subjects, three patients achieved complete responses (CRs), and one patient achieved partial responses (PR) (Mehta et al., 2022). FT825/ONO-8250 is another iCAR-T product of Fate Therapeutics that has been developed with the collaboration of “Ono Pharmaceutical.” It is undergoing assessment in a phase I clinical trial With or Without Monoclonal Antibodies for patients with advanced solid tumors (NCT06241456). FT825/ONO-8250 is a multiplex-CRISPR-edited iCAR-T product that harbors 7 genetic edits to promote their function. These edits include the targeted insertion of a novel anti-HER2 CAR into the TRAC locus, disruption of endogenous TCR, insertion of a CXCR2 transgene to promote cell trafficking to tumor sites, insertion of a high-affinity non-cleavable CD16A (hnCD16) transgene to enabling the cells to mediate ADCC, insertion of a TGFβ-signal redirection receptor to prevent the immunosuppressive effect of the tumor microenvironment, insertion of interleukin-7 receptor fusion protein to promote stemness of the iCAR-T cells, and disruption of CD38 to enhance durability of CAR-T cells in high oxidative stress tumor microenvironment (Hosking et al., 2023).

Fate Therapeutics is also evaluating three iCAR-NK products, FT522, FT576, and FT596, in early phase trials. FT596 is a multiplexed-edited iCAR-NK product that harbors three genetic edits, including a CD19 CAR containing NKG2D-2B4-CD3ζ signaling domains (designed to maximize cytolytic activity), an IL-15/IL-15Rα fusion protein (to enhance *in vivo* expansion), and hnCD16. In preclinical assessments, it has been demonstrated that the combination of FT-596 with rituximab led to better anti-tumor responses than conventional CD19 CAR-NK cells (Goodridge et al., 2019). In a multicenter Phase I clinical trial 20 patients with relapsed/refractory B-cell lymphomas and chronic lymphocytic leukemia were treated with FT596 in three regimen groups. After conditioning chemotherapy with fludarabine 30 mg/m^2^ and cyclophosphamide 500 mg/m^2^, patients received a single dose of FT596 as monotherapy (Regimen A), or in combination with rituximab 375 mg/m^2^ (Regimen B1) or obinutuzumab 1,000 mg/m^2^ (Regimen B2). Each single dose levels was single-dose levels between 30 and 900 million cells. The published clinical data reveal that administration of FT596 is well tolerated by all the 20 recipients with no reports of dose-limiting toxicity, GvHD, ICANS, or Grade ≥3 CRS. Among the 17 efficacy-evaluable patients, nine achieved objective responses, including seven complete responses. Seven out of nine patients with objective responses received a second cycle of treatment. The second cycle of treatment was well tolerated with no cases of CRS, ICANS, or GvHD and showed favorable efficacy with indications of continued therapeutic benefit (Bachanova et al., 2021). FT522 is this company’s next-generation CD19 iCAR-NK product that harbors more genetic edits than FT-596 to enhance its efficacy. The five genetic edits of FT-522 include a CD19 CAR, hnCD16, IL-15/IL-15Rα fusion protein, disruption of CD38, and an anti-4-1BB alloimmune defense receptor (ADR) (Williams et al., 2022). Administration of FT-522 with rituximab is currently under evaluation in a phase I trial (NCT05950334). FT576 is another multiplex genome-edited clinical-grade iCAR-NK product of Fate Therapeutics developed for individuals suffering from multiple myeloma. These cells are manipulated to express a BCMA CAR, hnCD16, an IL-15/IL-15Rα fusion protein, and elimination of CD38 expression. In preclinical studies, it has been demonstrated that Combined administration of FT576 with daratumumab results in stronger responses than monotherapy with FT576 or primary CAR-NK cells (Goodridge et al., 2020). This product is presently being assessed in a phase I dose-finding study. According to the recently released interim data of this trial, nine patients with relapsed/refractory multiple myeloma were treated with a single dose (on day 1) or multidose (on days 1 and 15) of FT576 as monotherapy or in combination with daratumumab. Administration of FT576 was well tolerated by patients, with no reports of GvHD, ICANS, CRS, or dose-limiting toxicity (Dhakal et al., 2022).

“Century Therapeutics” is another leading company in the field of CAR-iPSCs and is developing various iCAR αβ-T, iCAR γδ-T, and iCAR-NK products. CNTY-101, a multiplex genome-edited CD19 iCAR-NK product of this company, is currently under assessment in the phase I “ELiPSE-1” clinical trial for patients with CD19^+^ hematologic malignancies. These cells have two knock-out and four knock-in edits comprising the insertion of CD19 CAR, IL-15 transgene, safety switch, HLA-E, and disruption of β2M and CIITA (Krish Patel et al., 2024). According to the interim results of the ELiPSE-1 trial, 12 patients after the lymphodepletion regimen were treated with 10^7^, 30^7,^ or 100^7^ CNTY-101 cells at either Day 1 or Days 1, 8, and 15. Daily subcutaneous injection of IL-2 for a period of eight or 4 days after product administration was also performed for each patient.

Among the 12 patients treated with CNTY-101, no signs of GvHD, dose-limiting toxicity, ICANS, Induction of humoral immunogenicity, or Grade ≥3 CRS have been observed. Among the ten efficacy-evaluable subjects, the objective response rate and complete response rate were 40% and 30%, respectively. Following infusion, CNTY-101 cells quickly exited circulation and were detectable using cell-free DNA for up to 28 days. In two patients who received the second cycle of CNTY-101 without prior lymphodepletion the persistence of infused cells was not negatively affected, indicating the lower immunogenicity of CNTY-101 cells (Krish Patel et al., 2024).

## 7 Future prospects and conclusion

In this review, we have discussed how the current bottlenecks of CAR-T cell therapy are expected to be overcome by combining this field with iPSC technology. The use of iPSCs as the sources for generating CAR-modified cell products offers several advantages, including providing a potentially unlimited source for developing an off-the-shelf product, removing the issue of batch-to-batch inconsistency, ease of multiplexed genome editing, and allowing product re-administration (if needed). Although the preclinical results and published clinical data underscore the promise of iCAR-modified cells, this emerging field is not without challenges. There is little clinical data regarding the safety and efficacy of iCAR-cell products. Moreover, differentiating hematopoietic lineages, especially T cells, from iPSCs and producing iPSC-derived CAR-modified cells under cGMP and on an industrial scale remains challenging. Thus, the focus should be on conducting more preclinical and clinical studies in this field. While the clinical trials evaluate the efficacy and long-term safety of iCAR cells, preclinical studies should focus on elucidating the molecular mechanisms of iPSC differentiation to control and streamline this process and optimize it for industrial scale. Here we propose some Areas for Future Research:

- Optimizing Manufacturing Scalability: Manufacturing and scaling iCAR-T cells while ensuring cGMP compliance and maintaining cell quality is one of the main obstacles to the clinical translation of iCAR-T cells. The development of more efficient bioreactors and closed-system automation is crucial for scaling ICAR-T cell production. This can lead to enhanced reproducibility, reducing the risk of contamination, standardization of manufacturing protocol, and minimizing batch-to-batch inconsistency by reducing manual labor. The potential of artificial intelligence and machine learning can be harnessed for predictive modeling of cell growth patterns, optimizing the culture conditions and differentiation process, and real-time troubleshooting.
- Reducing the negative consequence of gene editing: Gene editing is an inevitable step for generating iCAR-T cells. This is performed to prevent GvHD, reduce immunogenicity, and enhance safety and efficacy. Nonetheless, gene editing, particularly when a multiplex strategy is employed, is associated with the risk of genotoxicity such as chromosomal abnormalities and off-target cleavages. Future studies should focus on optimizing the structure and mechanism of action of gene editing tools to reduce their genotoxicities. Long-term follow-up studies should be conducted to elucidate the long-term consequences of the edited genes.
- Reducing the production time and cost: Reducing the production timeline and costs is crucial to democratize access to this treatment option. Developing continuous automated and closed manufacturing systems, generating pre-validated and optimized IPSC cell lines and ZIP CAR technology can significantly help to overcome the issue of production time and treatment affordability.
- Expanding to Solid Tumors: Given the suboptimal efficacy of CAR-T cell therapy in solid tumors, subsequent studies should focus on developing novel strategies to overcome the current bottlenecks of CAR-T cell therapy in solid tumors
- Long-term Safety Studies: For validating the clinical applicability of these therapeutics conducting long-term safety assessment trials for monitoring any potential adverse event such as tumorigenicity is crucial.
- Platform-based Approvals: Platform-based approval allows to reuse of the same methodologies and facilities for multiple products, streamlining the clinical translation by reducing the time needed for regulatory review.

In conclusion IPSC-derived CAR-engineered cells hold transformative potential for oncology. By solving the above-mentioned hurdles, ICAR cells can reshape CAR-based immunotherapy by making it safer, efficacious, and accessible.
